# Flavonoids for gastrointestinal tract local and associated systemic effects: A review of clinical trials and future perspectives

**DOI:** 10.1016/j.jare.2025.01.014

**Published:** 2025-01-10

**Authors:** Xiaopeng Li, Enjun Xie, Shumin Sun, Jie Shen, Yujin Ding, Jiaqi Wang, Xiaoyu Peng, Ruting Zheng, Mohamed A. Farag, Jianbo Xiao

**Affiliations:** a Center of Nutrition and Food Sciences Hunan Agricultural Products Processing Institute Hunan Academy of Agricultural Sciences Changsha China; b School of Public Health Zhejiang University School of Medicine Hangzhou China; c National Clinical Research Center for Metabolic Diseases Metabolic Syndrome Research Center Department of Metabolism and Endocrinology The Second Xiangya Hospital of Central South University Changsha China; d Ausnutria Dairy Co., Ltd., Changsha 410200 China; e Pharmacognosy Department, Faculty of Pharmacy, Cairo University, Cairo 11562 Egypt; f Universidade de Vigo, Nutrition and Bromatology Group, Department of Analytical Chemistry and Food Science, Instituto de Agroecoloxía e Alimentación (IAA) – CITEXVI 36310 Vigo, Spain; g Research Group on Food, Nutritional Biochemistry and Health, Universidad Europea del Atlántico, Isabel Torres 21 39011 Santander, Spain

**Keywords:** Dietary flavonoids, Metabolism, Metabolic disorder, Health benefits, Clinical evidence

## Abstract

**Background:**

Flavonoids are naturally occurring dietary phytochemicals with significant antioxidant effects aside from several health benefits. People often consume them in combination with other food components. Compiling data establishes a link between bioactive flavonoids and prevention of several diseases in animal models, including cardiovascular diseases, diabetes, gut dysbiosis, and metabolic dysfunction-associated steatotic liver disease (MASLD). However, numerous clinical studies have demonstrated the ineffectiveness of flavonoids contradicting rodent models, thereby challenging the validity of using flavonoids as dietary supplements.

**Aim of Review:**

This review provides a clinical perspective to emphasize the effective roles of dietary flavonoids as well as to summarize their specific mechanisms in animals briefly.

**Key Scientific Concepts of Review:**

First, this review offers an in-depth elucidation of the metabolic processes of flavonoids within human, encompassing the small, large intestine, and the liver. Furthermore, the review provides a comprehensive overview of the various functions of flavonoids in the gastrointestinal tract, including hindering the breakdown and assimilation of macronutrients, such as polysaccharides and lipids, regulating gut hormone secretion as well as inhibition of mineral iron absorption. In the large intestine, an unabsorbed major portion of flavonoids interact with the gut flora leading to their biotransformation. Once absorbed and circulated in the bloodstream, bioactive flavonoids or their metabolites exert numerous beneficial systemic effects. Lastly, we examine the protective effects of flavonoids in several metabolic disorders, including endothelial dysfunction, MASLD, cardiovascular disease, obesity, hyperlipidemia, and insulin resistance. In conclusion, this review outlines the safety and future prospects of flavonoids in the field of health, especially in the prevention of metabolic syndrome (MetS).

## Highlights

• Elucidates the metabolism of flavonoids within the human body involving the intestine, gut microbiota, and liver.
• The multifaced functions of flavonoids in the gastrointestinal tract are provided, including regulating nutrients metabolism, gut hormone secretion, and reshaping gut microbiota.
• The associated systemic effects of flavonoids on metabolic homeostasis in peripheral organs are summarized.
• Dietary flavonoids benefit several disease progressions, and the safety and future prospects are also illustrated.

## Graphical abstract

**Figure:**
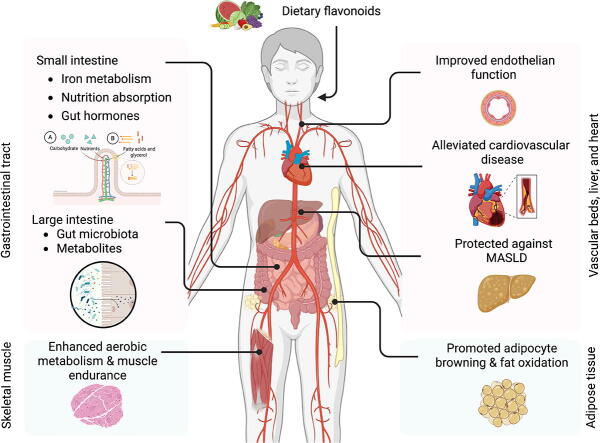

## Introduction

Flavonoids are secondary metabolites present in all vascular plants, comprising a vast collection of approximately 10,000 distinct compounds. Consequently, most vegetables, fruits, whole grains, and beverages such as fruit juices, coffee, wine, chocolate, and tea derived from plant extracts are abundant source of flavonoids. These compounds are known for their vivid pigments and the opulent hues found in a variety of fruits and vegetables. Their consumption has been linked to numerous health benefits, ranging from enhanced cardiovascular health to decreased susceptibility to metabolic dysfunction-associated steatotic liver disease (MASLD) and obesity [1], [2]. Dietary flavonoids undergo either limited absorption or significant metabolism in enterocytes and liver through phase I and phase II enzymatic processes [3]. Furthermore, they undergo extensive biotransformation by gut bacteria into a diverse range of novel chemical structures that can readily enter systemic blood circulation [4].

Experimental investigations in animal models have unequivocally demonstrated that flavonoids may offer therapeutic benefits in extending health and lifespan [5], [6], the alleviating metabolic syndrome (MetS) by influencing circadian rhythms, gut microbiota, and its metabolites [7], [8], [9], treating of cognitive dysfunction by modulating autophagy [10], protecting against cellular damage by suppressing ferroptosis [11], [12], and preventing cancer cells growth by activating apoptosis and pyroptosis [13], [14]. While animal studies provide valuable insights into the mechanisms and potential benefits of flavonoids, results may not always be directly applicable to humans due to differences in physiology, metabolism, and treatment responses between species. Therefore, comprehensive clinical trials to thoroughly assess the safety and efficacy of these therapies in humans are warranted, ultimately guiding direct clinical practice and enhancing public health.

Epidemiological studies have highlighted that a flavonoid-rich diet protects against chronic diseases such as hypertension, cardiovascular disease, MASLD, and diabetes [2], [15], [16], [17]. Excessive consumption of flavonoids among individuals with high cardiovascular risk is associated with a lower risk of overall mortality compared to those with lesser consumption [18]. Results from a prospective cohort study indicate that moderate regular consumption of flavonoids is negatively correlated with mortality from all causes, cardiovascular disease, and cancer, with the strongest correlation observed at daily doses of approximately 500 mg [19]. While epidemiological studies offer useful insights into the health benefits of flavonoids, they are fundamentally observational and unable to establish causative relationships. In contrast, clinical studies provide more stringent methodologies by accounting for confounding variables and implementing targeted interventions to evaluate the effects of flavonoids. Clinical research on flavonoids has been extensively investigated. From 2000 to 2020, flavanols were the most investigated class of polyphenols, followed by anthocyanidins and isoflavones [20]. The current analysis specifically examines the biological advantages of dietary flavonoids from a therapeutic perspective.

## Overview of flavonoids consumption worldwide

**Fig. 1:**
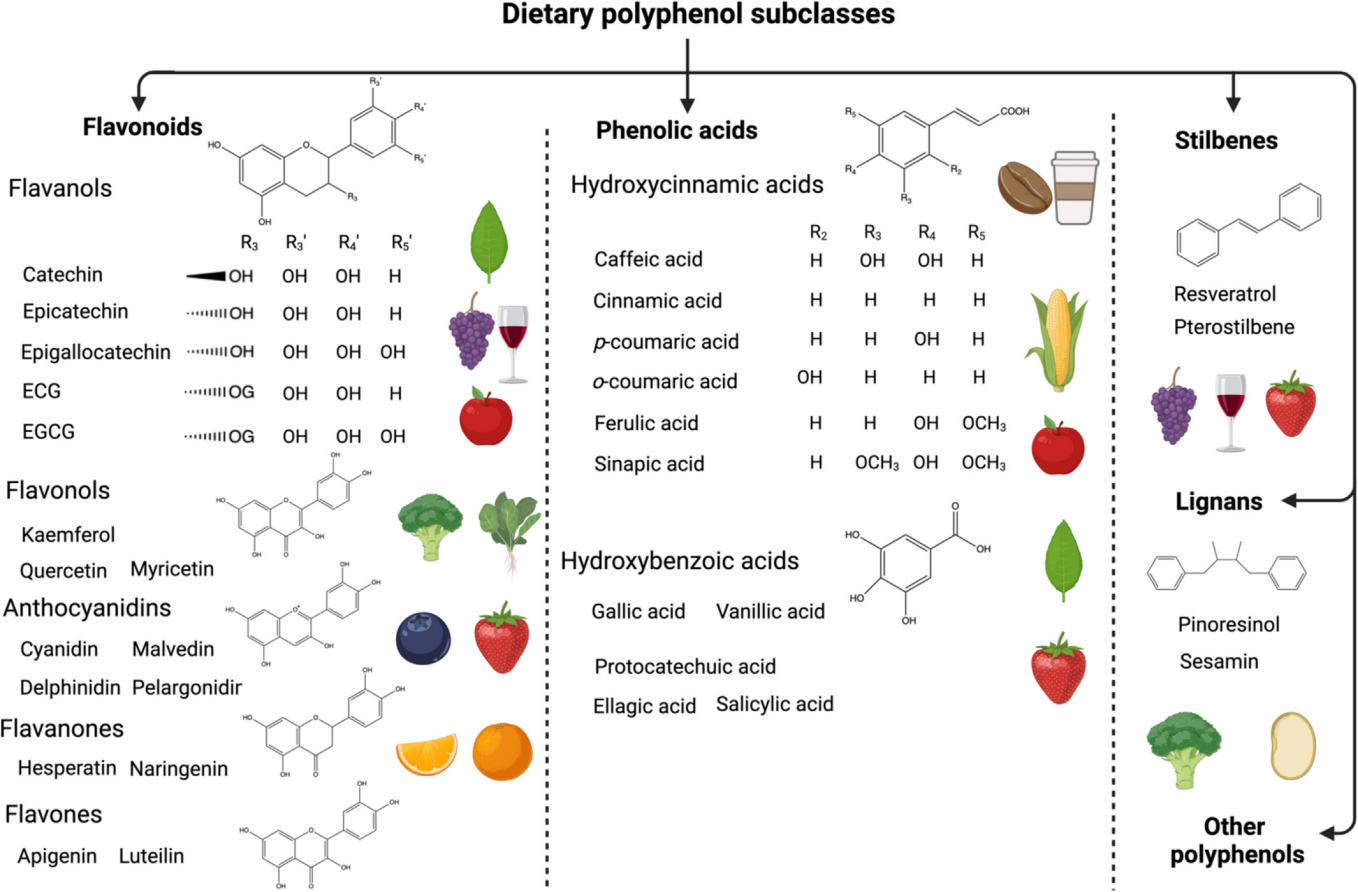
Dietary flavonoid subclasses, their basic chemical structure and typical dietary sources.

Based on the strength of the phenolic ring, polyphenols are classified into several categories, including flavonoids, phenolic acids, phenolic alcohols, and lignans (Fig. 1). Flavonoids are naturally occurring polyphenols characterized by a C6-C3-C6 skeletal structure, which consists of two aromatic rings connected by three carbon atoms. They are abundantly present in various foods, including major food crops, fruits, beverages, and certain types of wines. Notable flavonoid compounds, such as (−)-epigallocatechin gallate (EGCG) found in green tea, anthocyanins found in colored fruits, and procyanidins found in grape seed, have been extensively researched in the fields of food science and nutrition.

In the United States, the average daily polyphenol consumption remained nearly constant at 1666.3 mg/day between 2007 and 2016. The primary categories of polyphenols consumed were phenolic acids (1,005.6 mg/day) and flavonoids (379.1 mg/day). The food and beverage category accounted for 99.8 % of the flavonoid consumption, with coffee being the primary dietary source at 39.6 %, followed by beans at 9.8 % and tea at 7.6 % [21]. In Europe, the average total polyphenol consumption was highest in Denmark (1786 mg/day for men and 1626 mg/day for women) and lowest in Greece (744 mg/day for men and 584 mg/day for women). Phenolic acids were the primary contributors to polyphenol intake (52.5–56.9 %), except for men from Mediterranean countries and the UK health-conscious group where flavonoids prevailed as primary contributors (49.1–61.7 %) [22]. The main dietary sources of total polyphenols include coffee, tea, and fruits. The median, lower, and upper quartiles of flavonoid intakes among European teenagers were identified as 326, 167, and 564 mg/day, respectively. The primary dietary sources were fruit, which made up 23 % of the total, with apples and pears accounting for 16.3 %; chocolate goods representing 19.2 %; and fruit and vegetable juices covering 15.6 %. Flavonoids accounted for 75–76 % of the total polyphenol classes, whereas phenolic acids made up 17–19 % of the total, respectively [23]. In China, the median daily total flavonoid consumption is at 214.5 mg (ranging from 111.1 to 473.3 mg/day), indicating a notably lower intake of flavonoids compared to the United States and Europe. Flavonoids and phenolic acids represented the primary sources of the overall polyphenol intake, constituting 65 % and 30 % of the total consumption, respectively. Tea represents the primary source of flavonoids and phenolic acids in the diet, accounting for 72 % and 42 %, respectively [15]. The average daily phenolics consumption among university students in China was 1378  mg, with flavonoids accounting for 58.7 % of the total polyphenols consumed, followed by phenolic acids at 38.1 %. The most frequently consumed foods were vegetables, fruits, and cereal items [24]. Variation in food composition and dietary behaviors leads to distinct forms of flavonoid consumption. Flavan-3-ols and their oligomers are the predominant dietary flavonoid class and are extensively found in various food sources. Typically, tea is a prominent beverage in Asian countries, rich in flavan-3-ols, and serves as the primary source of these compounds. In contrast, in many Western countries, fruits are the main source of these compounds.

Flavonoids are consumed not as individual components but as intricate combinations, making it exceedingly difficult to determine which specific flavonoid is the most prevalent in our diet at any given time. Moreover, each category of flavonoids comprises numerous molecules with distinct substitution patterns, rendering the identification of the specific class of flavonoids contributing to maintaining nutritional balance becomes a complex task.

## The metabolism of flavonoids

**Fig. 2:**
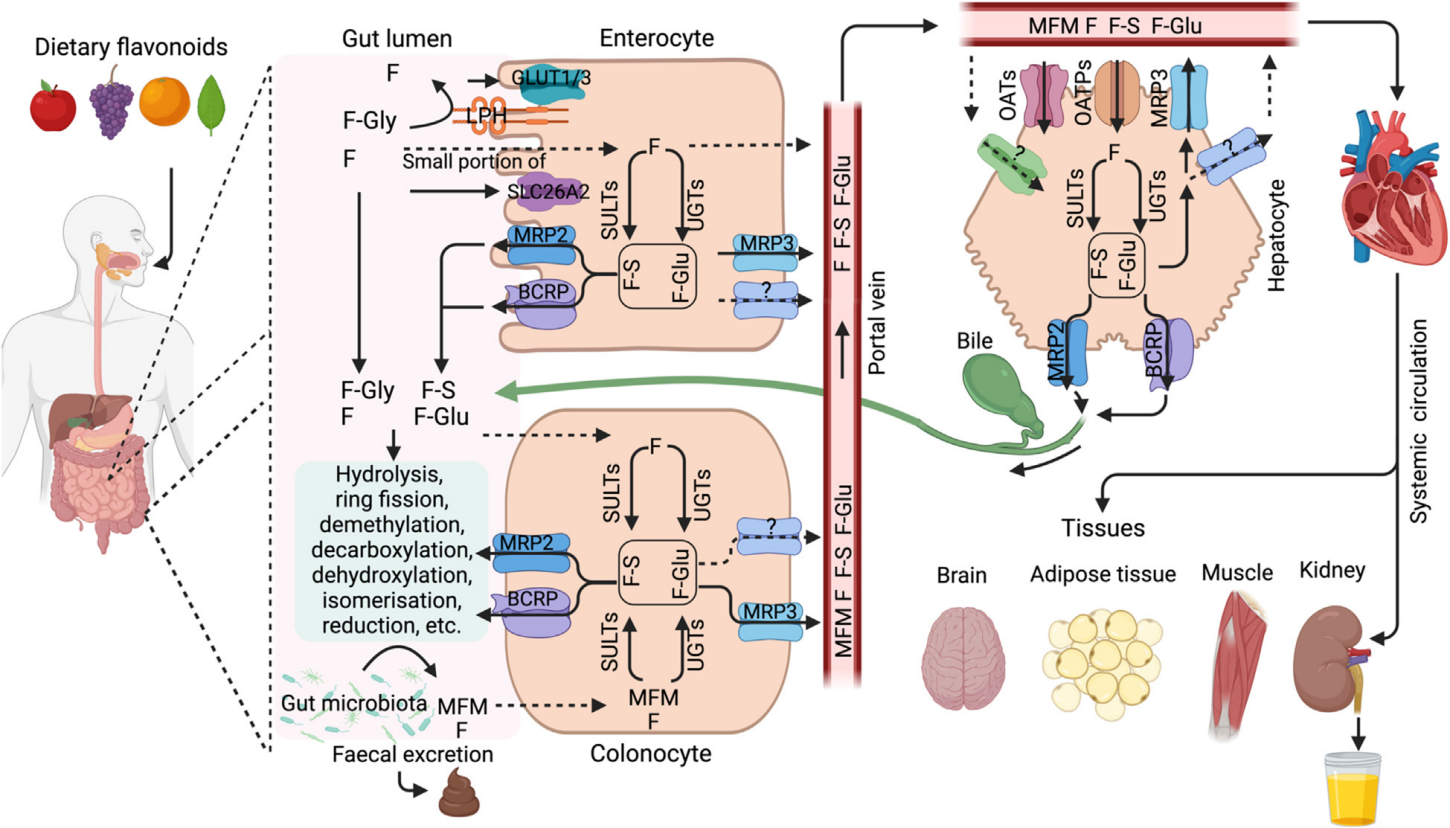
The metabolism of flavonoid in the intestine and liver. The hydrolysis of dietary flavonoid glycoside by LPH results in the formation of flavonoid. A quite tiny fraction of flavonoids manages to penetrate the intestinal epithelium and undergo phase II metabolic processes. The metabolites are transported into the portal vein for circulation or reach the intestinal lumen to undergo second metabolism by the intestinal microbiota. Most unabsorbed flavonoids are transported to and build up in the large intestine. Flavonoids undergo metabolism by gut microbiota through processes such as hydrolysis, ring fission, demethylation, dihydroxylation, and others. The phase II metabolites or microbial flavonoid metabolites (MFM), are also transported into the portal vein for arterial circulation. Within the liver, flavonoids can undergo phase II metabolism, and their resulting products are eliminated from the bile into the gut via the transporter MRP2/BCRP. BCRP, breast cancer resistance protein; F: flavonoid; F-Gly, flavonoid glycoside; F-Glu: flavonoid glucuronide; F-S: flavonoid sulfate; GLUT1/3, glucose transporter 1/3; LPH, lactase-phlorizin hydrolase; MFM, microbial flavonoids metabolites; MRP 2/3, multidrug resistance-associated protein 2/3; OATs, organic anion transporter; OATPs, organic anion transporting polypeptide; SULTs, sulfotransferases; UGTs, UDP-glucuronosyltransferase.

The metabolism of flavonoids, as well as their interaction with epithelial lipids have been previously summarized in several studies [25], [26], [27], [28]. Dietary flavonoids demonstrate robust stability in acidic pH solutions but exhibit limited chemical stability in neutral or alkaline environments. The metabolic pathways of flavonoids in the oral cavity, stomach, small intestine, and the large intestine under different pH conditions have been extensively studied [26], [29]. Here, we provided a concise overview of the role of hydrolase, transporters, and phase II metabolic enzymes in facilitating the absorption and metabolism of flavonoids (Fig. 2).

The structural characteristics of flavonoids—the parental skeleton structure, the quantity, and location of free hydroxyl groups, methoxy groups, and glycosides— determine the entry pathway of flavonoids into epithelial cells in the small intestine [30]. In the small intestine and colon, a multitude of enzymes and metabolites are involved in the rather intricate metabolism of flavonoid glycosides. Different processes govern the transport and metabolism of flavonoids with distinct structures in the gut and liver, and the metabolic processes of a single flavonoid may also include multiple pathways. Typically, the metabolism of flavonoids involves three crucial stages. The hydrolysis of flavonoid glycosides, which is considered the initial and crucial stage in the absorption of flavonoids, is facilitated by lactase-phlorizin hydrolase (LPH) found at the human intestine brush border [31]. Phase II metabolic enzymes, namely UDP-glucuronosyltransferase (UGTs), sulfotransferases (SULTs), and catechol-O-methyltransferase (COMT), are the primary enzymes responsible for the metabolism of flavonoids in living organisms [32]. UGTs are the primary contributors, followed by SULTs and COMT [33]. In contrast, the phase I metabolic pathway, which is facilitated by cytochrome P450 (CYPs), has a relatively insignificant impact. Furthermore, the interaction between transporters, such as breast cancer resistance protein (BCRP) and multidrug resistance-associated protein (MRPs), and phase II enzymes (UGTs and SULTs), is crucial for the specific distribution of flavonoids, particularly in the enterohepatic circulations [27], [34].

### Absorption and metabolism in the small intestine

LPH, which is found in the small intestine, is a protein that is attached to the cell membrane and is capable of breaking down flavonoid glycosides in the gut lumen. Following the selective inhibition of LPH using an *in situ* intestinal perfusion system, the hydrolysis of quercetin-3-glucoside was reduced by 67 %, and concurrent with 75 % less quercetin serum level. This indicates that the hydrolysis by LPH plays a significant role in the intestinal absorption of quercetin-glucosides [35]. Besides, the human gut microflora mostly comprises approximately 400 known bacterial species, which produce various hydrolases, including α-arabinofuranosidase, α-fucosidase, β-fucosidase, β-glucosidase, β-glucuronidase, and α-rhamnosidase [36]. These enzymes facilitate the hydrolysis of several flavonoid glycosides. When incubated with microflora derived from human faeces, flavonoid glycosides can undergo a conversion into their corresponding aglycones. In addition to hydrolases, the human intestinal microflora also contains several other enzyme systems capable of breaking down flavonoids into smaller constituents, primarily as phenolic acids [25], [37].

Lipophilic flavonoid aglycones such as apigenin, kaempferol, naringenin, and genistein can be readily absorbed by intestinal epithelial cells via passive diffusion [38]. However, the absorption of specific flavonoids, such as morin, as along with some flavonoid glycosides (e.g., baicalin, diosmetin-7-O-glucopyranoside, isorhamnetin-3-O-rutinoside, and daidzin), may also involve efflux processes [39]. The precise active transport mechanisms responsible for flavonoid absorption in the colon are not yet fully identified, as this process encompasses several transport systems operatively. For instance, the transportation of malvidin-3-O-glucoside decreased by ca. 25 % in human gastric epithelial cells (MKN-28) upon silencing glucose transporter 1 or 3 (*GLUT1* or *GLUT3*), and by 45 % when both transporters were simultaneously inhibited [40]. These findings indicate that GLUT1 and GLUT3 are involved in the recognition and uptake of malvidin-3-O-glucoside. The specific transporters responsible for the transportation of other flavonoids remain currently unknown, and have yet to be cloned. One study revealed that some transporters facilitated the absorption of EGCG. For example, *Xenopus laevis* oocyte overexpressing the transporter SLC26A2 exhibited EGCG absorption rate that was 4.8 times greater than that of the control oocytes. Moreover, the CHO-K1 cells that consistently expressed SLC26A2 absorbed EGCG at a rate 1.8 folds higher than that of the control cells. This enhancement was reduced by 15 % when exposed to a SLC26A2 inhibitor [41]. These findings indicated that the transporter SLC26A2 plays a role in the uptake of EGCG. However, several additional transporters and unidentified absorption processes have to be ascertained for other catechins.

### Phase II and phase I metabolism

Following absorption into the intestinal epithelial cells as aglycones, flavonoids undergo extensive metabolism facilitated by UGTs, SULTs, COMT, and CYPs in the gut and subsequently in the liver, a process known as the 'first-pass metabolism'. Considering the presence of phenolic hydroxyl groups in flavonoids, UGTs are recognized as the critical players in the degradation of flavonoids, with SULTs and COMT contributing significantly as well [42]. For example, EGCG undergoes metabolism to glucuronides mostly by variants of the human liver and intestine UGT enzymes (1A1, 1A3, 1A8, 1A9). Typically, conjugation takes place at the hydroxyl groups on C3′ or C4′ of the B ring and C5 or C7 of the A ring [25].

Phase I metabolism by CYPs also influences the fate of some flavonoids. The CYP superfamily is a vast and heterogeneous collection of enzymes, with just around a dozen enzymes specific to the CYP 1, 2, and 3 −families being accountable for the metabolism of most medicines and other xenobiotics. Flavonoid oxidation via CYPs involves both hydroxylation and demethylation [43]. In addition to the liver, which exhibits the highest expression, CYP activity is also present in multiple non-hepatic organs, including the small intestine mucosa, lung, and kidney. *In vivo* studies suggest that flavonoids are more readily conjugated by UGTs, SULTs, and COMT than metabolized by CYPs [44], [45].

### Key drug transporters involved in the fate of conjugated flavonoids

Following the intracellular glucuronidation or sulfation of flavonoids by UGTs or SULTs, several drug transporters including MRPs, BCRP, organic anion transporters (OATs), organic anion transporting polypeptide (OATPs), P-glycoprotein (P-gp), and peptide transporters (PEPTs) have been identified as mediators for the transportation of flavonoid −glucuronides (F-Glu) and −sulfates (F-S) [27]. In the liver, solute carrier (SLC) transporters, including OATs and OATPs, facilitate the uptake of flavonoid conjugates from the bloodstream [46], [47]. MRP2 and BCRP in the apical domain, along with MRP3 in the basolateral domain contribute to the efflux of the flavonoid conjugates from the liver into the gut lumen or systemic circulation. During intestinal digestion, SLC26A2 and GLUTs may assist in the uptake of flavonoids from the gut lumen [41], while MRP3 and other transporters facilitate the release of flavonoid conjugates into the bloodstream [48].

## The multifaceted roles of flavonoids in the gastrointestinal tract

Upon consumption of flavonoids, the unique structures formed by the hydroxyl and carbonyl groups of flavonoids enable effective interaction with the carboxyl groups of glutamic acid, the hydroxyl of serine, aspartic acid, or histidine in the side chain of the enzyme active center [49]. This interaction can inhibit the activity of polysaccharide hydrolase and lipase, or disrupt the function of associated transporters, thereby influencing the digestion and absorption of carbohydrates and fats [49], [50], [51], in addition to micronutrient absorption and regulation [52]. Furthermore, flavonoids can interact with Fe^2+^/Fe^3+^ ions to form complexes, alter the composition of intestinal microbiota, and potentially participate in other processes that impact human health [9], [53].

### Flavonoids modulate iron homeostasis

#### Iron metabolism

**Fig. 3:**
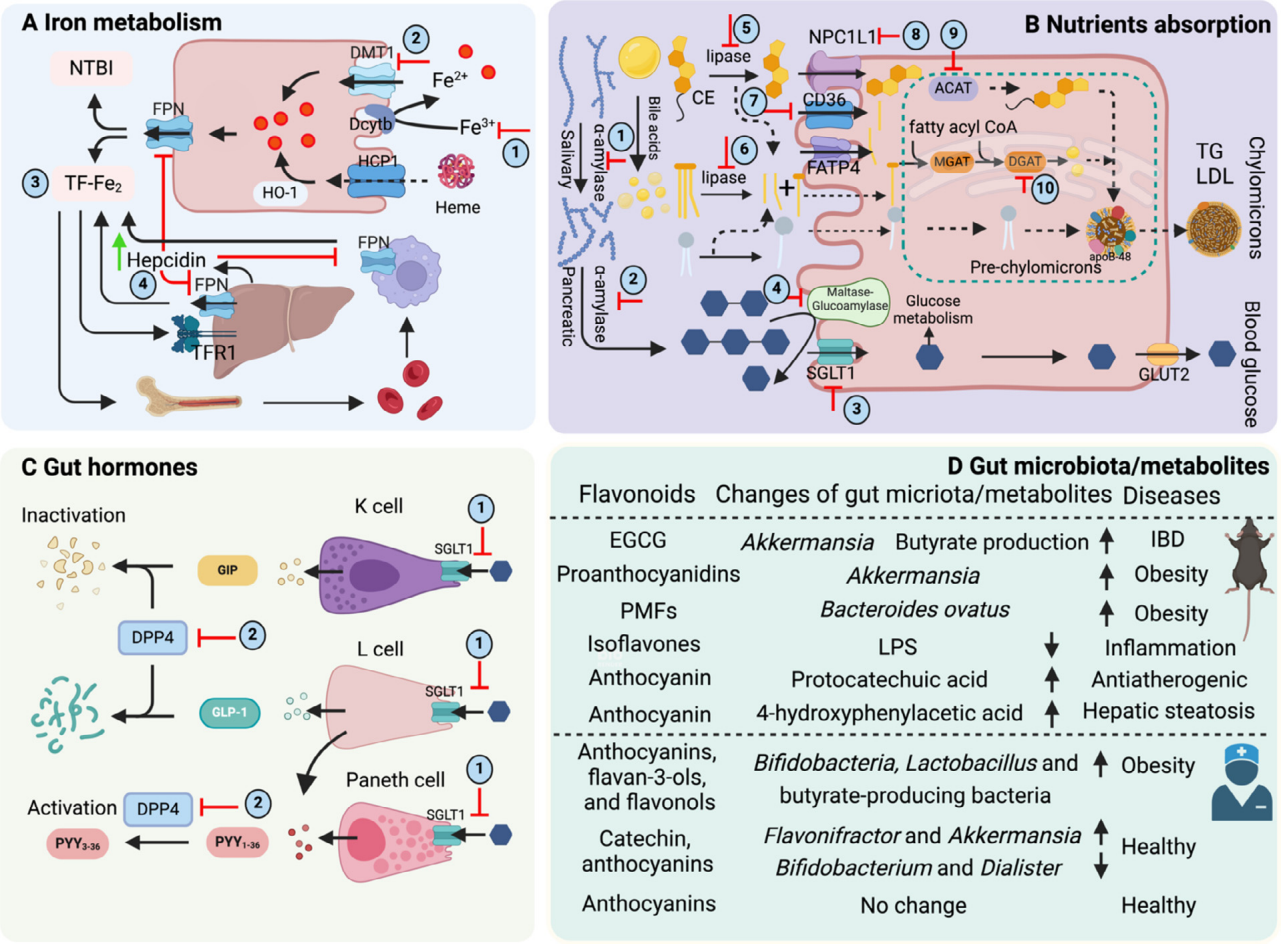
**The multifaceted roles of flavonoids in the gastrointestinal tract. A, Iron metabolism.** Dietary flavonoid can bind Fe^3+^/Fe^2+^ to form complex (1), inhibit DMT1 (2), the transferrin saturation (3), and hepcidin expression (4) to regulate iron homeostasis. **B, Intestinal glucose/lipid uptake and transport.** Dietary flavonoid can suppress amylase (1–2), SGLT (3), and maltase/glucoamylase activity (4), thus regulating glucose metabolism. Besides, dietary flavonoids have been reported reduced lipase activity (5–6), inhibited CD36/NPC1L1(7–8), and lipid biosynthesis ACAT/DGAT (9–10). **C, Gut peptides secreted by enteroendocrine cells (EECs)**. The apical membrane of EEC is responsible for nutrients sensing, which necessitates the uptake of glucose and Na^+^ ions into the cell by SGLT-1, which in turn triggers vesicle fusion and the release of gut peptides. Dietary flavonoids suppress the metabolic breakdown of glucose and lipids, therefore potentially impacting the release of GLP-1/GIP. Furthermore, consumption of dietary flavonoids has been demonstrated to suppress the activity of dipeptidyl peptidase 4 (DPP4). **D, The interaction between flavonoids and gut microbiota.** The regulation of certain gut flora (*Akkermansia* and *Bacteroides ovatus*) or metabolites (reduced LPS, generating protocatechuic acid, and 4-hydroxyphenylacetic acid) by dietary flavonoids in mouse models leads to improvements in obesity, hepatic steatosis, and inflammation. The regulation of gut flora by flavonoids in human studies is a more intricate phenomenon. Several flavonoids do not demonstrate any substantial impact. ACATs, acyl-CoA: cholesterol acyltransferases; ApoB-48, apolipoprotein B-48; CE, cholesteryl esters; DCTYB, duodenal cytochrome *b*; DGATs, diacylglycerol acyltransferases; DMT1, divalent metal transporter 1; DPP4, dipeptidyl peptidase 4; EGCG, (−)-epigallocatechin gallate; FATP4, fatty acid transport protein 4; FPN, ferroportin; GLP-1, glucagon-like peptide 1; GIP, glucose-dependent insulinotropic polypeptide; GLUT2, glucose transporter 2; HCP-1, heme carrier protein 1; HO-1, heme oxygenase 1; HIF, hypoxia-induced factor; IBD, inflammatory bowel disease; LPS, lipopolysaccharide; PMFs, polymethoxyflavones; PYY, peptide YY; RBCs, red blood cells; MGATs, monoacylglycerol acyltransferases; NPC1L1, Niemann-Pick C1-like 1; NTBI, non-transferrin bound iron; SGLT1, Na^+^-D-glucose cotransporter 1; TGs, triacylglycerols; TFR1, transferrin receptor 1. (For interpretation of the references to colour in this figure legend, the reader is referred to the web version of this article.)

The systemic and hepatic iron hemostasis inside the body and its role in liver diseases has been previously reviewed [54]. Following the consumption of iron, it undergoes reduction by duodenal cytochrome *b* (DCYTB) and is subsequently transported into the enterocytes *via* divalent metal transporter 1 (DMT1). Heme oxygenase-1 (HO-1) is responsible for the degradation of dietary heme in enterocytes. Following its exportation by ferroportin (FPN), Fe^3+^ binds to transferrin (diferric transferrin, TF-Fe_2_), migrates to tissues, and is primarily utilized in the production of new red blood cells. In order to recycle iron, macrophages enzymatically break down aged red blood cells (RBCs) [55]. Furthermore, the liver plays a critical role in regulating iron metabolism by generating and releasing hepcidin, the primary regulator of iron homeostasis, thus ensuring systemic iron balance. Transferrin-bound iron (TBI) and non-transferrin-bound iron (NTBI) are acquired by hepatocytes through transferrin receptor 1 (TFR1) and SLC39A14, respectively [56]. Within hepatocytes, iron is sequestered in ferritin and released by FPN. The binding of flavonoids to iron results in the formation of a non-transportable complex, thereby reducing the absorption of iron in the body [57] (Fig. 3**A**). Consequently, this can heighten the susceptibility to iron insufficiency. Individuals who are already susceptible to iron deficiency face an elevated risk when consuming excessive quantities of green tea extract or EGCG [58].

#### Inhibition of iron absorption and remodeling of iron regulation by dietary flavonoids

In order to understand the underlying mechanisms, we provide a summary of the effects of flavonoids on iron metabolism in animal or cell models. Oral quercetin dramatically reduced iron absorption, while intraperitoneal quercetin significantly decreased levels of duodenal DMT1, Dcytb and FPN in rats [59]. Dietary flavonoid myricetin significantly inhibits the expression of hepcidin (the primary regulator of iron homeostasis) both *in vitro* and *in vivo* [60]. The tiny chemical genistein enhances the binding of Stat3 to the hepcidin promoter, therefore increasing hepcidin expression in human hepatocytes [61]. Following treatment with the berry-extract, there were significant decreases in *DMT1* and *HFE* mRNA expression in human intestinal Caco-2 cells [62]. The administration of rutin led to a notable decrease in the expression of hepatic ferritin protein expression and serum transferrin saturation in mice lacking the transferrin receptor 2 (*Tfr2*) gene. Furthermore, detectable patterns of reduced iron levels were observed in the liver and blood, as long as an increase in serum unsaturated iron binding capacity [63].

Some reviews have summarized the relationship between flavonoids and iron homeostasis [57], [64]. Table 1 summarizes several clinical studies on the inhibition effects of flavonoids on iron absorption. For example, consumption of 200–300 mL of tea effectively reduces iron absorption in healthy individuals [65]. Unlike other flavonoids, isoflavones have less effect on serum iron levels [66], [67], [68]. A significant factor influencing the inhibitory impact of compounds on iron absorption from the diet is the presence of iron-binding galloyl groups, while the phenolic catechol groups appear to have less influence [52]. Unfortunately, we were unable to find human clinical trials examining the impact of flavonoids on the absorption of heme iron warranting for future studies. However, the inhibitory effects of EGCG, green tea extract, and grape seed extract on intestinal heme iron absorption in human intestinal Caco-2 cells were manifested by inhibited basolateral iron export [69]. This finding suggests that flavonoids have the potential to hinder heme iron absorption in humans, though yet to be compared for its different subclasses.

**Table 1:** Flavonoids and iron absorption.

Flavonoids	Participants	Test	Inhibition	References
Procyanidins	Hereditary hemochromatosis (n = 20) anddysmetabolic iron overload syndrome (n = 20)	100 mg procyanidins + a standardized test meal (43 mg iron)	No effect	[77]
Isoflavones	Postmenopausal women (n = 14)	84.6 mg Isoflavones + 5.7 mg iron	No effect	[70]
Flavonols and catechins	Iron deficiency anemia (n = 10) and healthy women (n = 10)	Tea (1 or 2 cup) + 3 mg iron	49–67 %	[295]
Flavonols and catechins	Healthy women (n = 12)	200 mL tea + 4 mg iron	37 %	[65]
Flavonols, procyanidins, and catechins	Hereditary hemochromatosis (n = 14)	Test meal contained 8 mg iron; drinks consisted of 10 mg Fe	40 %	[76]
Herb Tea	Healthy mothers (n = 17) and children (n = 17)	Tea + 4 mg iron	50–70 %	[296]

Flavonoids also modulate iron metabolism in the body. Isoflavones decreased the total TS by nearly 3.8 % [66], [70] and raised the haemoglobin concentration by around 12.9 g/L in healthy subjects [66], [71], [72]. Nevertheless, excessive consumption of green tea can result in iron deficient anemia. For example, after consuming over 1500 mL of tea daily for twenty years, a patient displayed iron deficiency, as evidenced by markedly reduced levels of serum ferritin (1.6 ng/mL), serum iron (10 μg/dL), and transferrin saturation (1.95 %), along with an increased total iron-binding capacity (513 μg/dL) [58]. Excessive consumption of tea over a long period of time may lead to abnormal iron metabolism and anemia, however, moderate intake of tea in daily life does not cause iron deficiency [58], [73]. A *meta*-analysis reported that flavonoids (silymarin and quercetin) reduce the concentration of SI, TS, and ferritin concentrations in patients with β-thalassemia, albeit with any effect on haemoglobin concentration [71], although in normal cases increase in hemoglobin was observed in response to flavonoids.

Mostly, hemochromatosis (HH) is attributed to genetic mutations, with C282Y and H63D mutations of the *HFE* gene being the primary etiological factors in hereditary hemochromatosis. Additionally, other genetic abnormalities such as *HJV* (G320V), *Hepcidin* (R56X), *TFR2* (Q317X), *FPN* (A77D), and *H-ferritin* (A49U) result in abnormal iron distribution and the surplus accumulation of iron in organs, particularly the liver, heart, and pancreas [74], [75]. The therapeutic interventions include intravenous blood transfusion and chelation therapy with desferoxamine and deferiprone. Consumption of adequate amount of food fortified with flavonoids during patient therapy may be beneficial for managing the disease. In individuals with type I HH, the consumption of a 300 mg flavonoid supplement with black tea powder, cocoa powder, and grape juice extract reduced fractional iron absorption by 40 % when the dietary iron intake was between 8.0 and 10.0 mg [76]. Conversely, supplementation with 100 mg of procyanidin did not have a significant effect on iron absorption (43 mg iron in diet) in individuals with dysmetabolic iron overload syndrome or in those with HH [77]. Comparison among results is not possible as different forms of flavonoids were administered to reveal a dose effect**.** In general, both animal and clinical studies provide evidence that flavonoids effectively hinder the absorption of iron ions and regulate iron ion metabolism. The use of flavonoids as a treatment for patients should be addressed with considerable caution, especially if administered over a long period of time.

Thalassemia comprises a collection of hereditary illnesses caused by abnormalities in the synthesis rate of globin chains. Individuals with thalassemia may accumulate excessive iron in their systems, either due to the condition itself or as a result of frequent blood transfusions [78]. In β-thalassemia, iron overload can cause damage to various organs, particularly the liver and heart. For organ iron excess to be avoided or reversed, iron chelation therapy is essential [79]. 500  mg/day quercetin for 12 weeks ameliorate the iron status in thalassemia major by reducing high sensitivity C-reactive protein, iron, ferritin, and transferrin saturation, and increase transferrin significantly [80]. 280 mg silymarin daily for 12 weeks treatment resulted in a negative change in the serum iron, ferritin levels and a positive change in the total iron‐binding capacity levels [81]. In the combination of desferrioxamine and silymarin (420 mg for 9 month) group, serum ferritin, iron, and total iron-binding capacity values were considerably lower than those of the desferrioxamine group. Following a 9-month course of treatment, patients receiving silymarin medication also showed a significant drop in serum levels of soluble transferrin receptor and hepcidin [82]. A similar study in Egyptian child with β-thalassemia major also showed serum ferritin and iron were significantly lower in combination of deferiprone and silymarin (420 mg for 9 month) group than deferiprone group, while no statistically significant differences in serum creatinine, blood urea, ALT and AST [83]. There is less literature related to other flavonoids for the prevention and treatment of thalassemia, and this may be related to the efficiency with which flavonoids affect iron metabolism.

### Flavonoids inhibit polysaccharides hydrolysis and glucose transport

Salivary α-amylase initiates starch digestion in the mouth by hydrolyzing the α 1–4 glycosidic bonds of starch, resulting in the formation of small polysaccharide fragments or oligosaccharides. In the stomach, the low pH renders salivary α-amylase inactive. The pancreas releases a different form of α-amylase into the small intestine, which persists in facilitating the breakdown of the biological process. The enzymes of the intestinal brush border primarily further break down the maltose, maltotriose, and dextrin produced by pancreatic α-amylase into glucose. Glucose is subsequently uptaken by the transporters sodium-coupled glucose transporter 1 (SGLT1) and GLUT2 [84] (Fig. 3**B**).

*In vitro* and *in vivo* animal assays have demonstrated that flavonoids have a diverse function in the process of starch digestion, mainly *via* inhibiting digestive enzymes (α-amylase and α-glucosidase) and glucose GLUT1 [85], [86]. Flavonoids exhibited varying levels of inhibition and selectivity towards two enzymes derived from the tissues of the mouse pancreas and small intestine, specifically α-amylase and α-glucosidase [87], [88]. Flavanols major form in onion have been shown to selectively block the intestinal SGLT1. Interestingly, these compounds do not exhibit any anti-hyperglycemic actions *in vivo* in normoglycemic mice and human volunteers [89]. Sun et al. reported that 8 flavonoids, namely apigenin, kaempferol, eupatilin, luteolin, hispidulin, isosinensetin, sinensetin, and nobiletin, showed substantial inhibition (>50 %) on GLUT1 in HEK293T cells [51].

The potential impact of flavonoids on glucose metabolism in humans is highly significant (Table 2). The administration of a 2.8 g apple extract capsule (phlorizin and quercetin) 30 min before the oral glucose tolerance test (OGTT) was shown to decrease postprandial blood glucose levels in healthy lean men [90]. This effect may be attributed, in part, to the suppression of glucose transport by SGLT1 and reduced renal glucose reabsorption [90]. Administration of 1200 mg of apple flavonoids, or 600 mg of apple flavonoids combined with 600 mg of blackcurrant anthocyanins, resulted in a significant decrease in plasma glucose levels, postprandial insulin, C-peptide, and glucose-dependent insulinotropic polypeptide (GIP) levels following the consumption of a mixed carbohydrate meal. This suggests that flavonoids inhibit the breakdown of starch or sucrose, as well as the transportation of glucose [91]. There were no significant differences in peak plasma glucose levels between subjects who had either water or 25 g of cacao polyphenol-rich chocolate (epicatechin and procyanidins) mixed with water in OGTT [92]. Supplementing with a single 46 g dose of grape powder (cyanidin and malvidin) did not alter their postprandial responses in obese individuals [93], possibly due to the inadequacy of the polyphenol dosage to elicit these effects.

**Table 2:** Flavonoids and postprandial glucose.

Flavonoids	Participants	Test	Postprandial glucose	References
Epicatechin and procyanidins	48 healthy participants	Oral glucose tolerance test	No change	[92]
Cyanidin and malvidin	25 obese subjects	Breakfast + grape power	No change	[93]
Anthocyanins, delphinidins	10 healthy participants	Rice and berry extract	Decreased	[297]
Quercetin and isorhamnetin	10 healthy men	Oral glucose tolerance test	Decreased	[90]
Anthocyanins and flavan-3-ols	20 healthy participants	A starch and sucrose meal + flavonoid	Decreased	[120]
Catechin-rich tea	17 healthy participants	70 % from carbohydrates + green tea twice	Decreased	[122]
Hesperidin	10 healthy participants	Orange juice + hesperidin	Decreased	[298]
Apple extract flavonoid	30 healthy participants	Starch meal + apple extract flavonoid	Decreased	[91]
Anthocyanin	17 healthy men	Starch meal + anthocyanin	Decreased	[299]
Polymerized flavonoids + catechins	15 health females	Fat meal + Oolong tea flavonoids	Decreased	[107]

Flavonoids exhibit favorable chemical characteristics for inhibiting α-amylase, namely hydroxylation at the 3′- and 4′- positions of the B ring and at the 5-, 6-, and 7-positions of the A ring [94]. Furthermore, the inhibitory effect of flavonoids under investigation on α-amylase is diminished by the methylation and glycosylation of –OH groups in the A and B rings, as well as the presence of a –OH group at the 3-position of the C ring. The effective inhibition of α-glucosidase is facilitated by the presence of –OH groups at the 3-position of the C ring, at the 3′-, 4′-, and/or 5′- positions of the B ring, and at the 5-, 6-, 7- and/or 8-positions of the A ring. The inhibitory action of flavonoids was diminished by glycosylation, particularly at the 3-position of the C ring and the 7-position of the A ring [95], and suggestive that algycones are more potent than corresponding glycosides in lowering sugar level. Overall, flavonoids exhibit potential antidiabetic effects by regulating the activity of both α-amylase and α-glucosidase enzymes.

### Flavonoids regulate lipid absorption

#### Digestion, absorption, and transportation of lipid

Dietary lipids, including triacylglycerols (TAGs), cholesteryl esters (CE), and phospholipids (PLs), as well as endogenous lipids derived from bile (PLs and cholesterol), are broken down by pancreatic enzymes inside the intestine. This process yields fatty acids (FAs), monoacylglycerols (MAGs), cholesterol, and lysophospholipids (LPLs). FAs and MAGs can be taken up either by the concentration gradient or with the assistance of transporters such as CD36 and fatty acid transport protein 4 (FATP4) [96], [97]. The process of cholesterol absorption is facilitated by the protein Niemann-Pick C1-like 1 (NPC1L1), while LPLs are absorbed through passive mechanisms [98]. Once absorbed by enterocytes, the breakdown products of lipids are converted back into their original form through the action of a group of proteins, such as monoacylglycerol acyltransferases (MGATs), diacylglycerol acyltransferases (DGATs), and acyl-CoA: cholesterol acyltransferases (ACATs). These enzymes function collaboratively to reassemble the lipids into TAGs, CEs, and PLs [99]. Subsequently, the intestinal lipids are assembled into pre-chylomicrons in conjunction apolipoprotein B-48 (apoB-48) or retained as lipid droplets within the cells. Fully developed chylomicrons are released through exocytosis and transported into the lymphatic system. From there, droplets enter the bloodstream, which transports them to muscle and adipose tissue [100]. Fatty acids are liberated by lipoprotein lipase in the capillaries of these tissues. In muscle, these fatty acids are then oxidized to provide energy, while in adipose tissue, they are re-esterified and stored as triacylglycerols. After triglycerides are absorbed, most of the chylomicrons are delivered to adipose tissue and skeletal muscle with only 20 % of them sent to the liver, where they are cleared within a few hours after consuming a meal containing fat. In the endogenous pathway, lipids that are produced or packaged in the liver are transported to peripheral organs by very-low-density lipoprotein (VLDL) [101]. The process of extracting lipids from VLDL, while also losing some apolipoproteins, progressively transforms a portion of VLDL into low-density lipoprotein (LDL). LDL then transports cholesterol to tissues outside the liver or returns it back to the liver (Fig. 3**B**).

#### Flavonoids effect on lipid absorption and metabolism

*In vitro* investigations have revealed that flavones showed more potent inhibitory action against lipase compared to other flavonoids. With an IC_50_ value of 99 ± 11 μM, luteolin showed the most potent inhibitory action against lipase, followed by quercetin and baicalein [50]. Both naringenin and hesperetin decrease the availability of lipids for the assembly of apoB-containing lipoproteins, an effect mediated by reduced activities of ACAT1 and ACAT2 in HepG2 cell study [102]. Flavonoids have also been shown to exhibit specific inhibitory effects on DGAT, the enzyme responsible for triglyceride production in the glycerol phosphate pathway [103].

Promising findings from human trials indicated that the consumption of flavonoids may have an impact on the digestion and absorption of lipids **(**Table 3). Administration of 750 mL of tea rich in catechins enhanced the elimination of lipids into the feces and reduced the levels of triglycerides in the bloodstream among healthy individuals consuming 38 g of lipids (19 g lipids each, taken within 30 min after lunch and dinner) over 10 days [104]. Following the consumption of a beverage containing 55 mg of black tea flavonoid three times a day for 10 days in healthy people, the total lipid excretion in stool samples increased by 24.6 %. The fat intake for males was 84.5 g, while for females it was 68.0 g [105]. The ingestion of EGCG at a dosage of 1450 mg decreased the levels of circulating catecholamines, but did not affect metanephrine, glucose, or lactate levels. Additionally, the peak rate of lipid oxidation was 32 % lower in healthy male participants [106]. No significant changes in early-phase postprandial triacylglycerol levels were observed in healthy persons who consumed 40 g of fat when exposed to polymerized oolong tea flavonoids at a dosage below 150 mg, regardless of the presence or absence of 50 mg caffeine and 63 mg catechins [107]. Comparison of different tea types on lipid metabolism should be investigated clinically considered the major differences in flavonoid composition and levels. The consumption of blueberry extract containing 364 mg of anthocyanin and 879 mg of phenolics, together with a high-fat (64.5 g) and high-sugar (84.5 g) meal, did not have any impact on the levels of serum LDL-C and triglycerides (TG) in individuals with MetS (body mass index, BMI = 31.4) [108]. Therefore, individual flavonoids exhibit varied inhibitory effects on lipase, necessitating a specific quantity for effectively suppression of lipid breakdown.

**Table 3:** Flavonoids and lipid absorption.

Flavonoids	Participants	Test	Lipid excretion or Blood lipid profile	References
Polymerized flavonoids + catechins	15 healthy adults	40 g lipid + 163 mg flavonoids	No change in blood lipid profile	[107]
Flavanols and proanthocyanidins	18 type 2 diabetes	High fat meal + 20 g cocoa extracts	No obvious changes	[153]
Catechins + monoglucosyl hesperidin	36 healthy adults	4 weeks 165 mg monoglucosyl hesperidin + 387 mg catechin	Significantly decreased fasting serum TG	[250]
Polymerized flavonoids + catechins	12 healthy participants	38 g lipid + 374 mg flavonoids + catechins	Increase fecal lipid	[104]
Theaflavins and thearubigins	25 healthy participants	55 mg black tea	Increase fecal lipid	[105]
Blueberry anthocyanin	45 participants with MetS	Energy-dense drink + 364 mg anthocyanin	Lowering cholesterol, and improving HDL-C	[108]

### Flavonoids influence gut hormone (GLP-1/GIP/GYY) secretion

Most GIP is produced in enteroendocrine K cells located in the duodenum and jejunum. Glucagon-like peptide-1 (GLP-1) is classified as an incretin hormone predominantly secreted by the L-cells in the intestinal tract (Fig. 3**C**). GIP, or GLP-1, is a member of the incretin class of molecules that trigger the release of insulin when ingested orally. The pharmacological effects of GIP and GLP1 are mediated by the GIP receptor (GIPR) and GLP1 receptor (GLP1R). GIP/GLP-1 receptor agonists have demonstrated a superior improvement effect in type 2 diabetes (T2D) [109]. A number of reviews have provided a concise overview of the impact of GIP and GLP-1 on appetite regulation, inflammation, adipose tissue, MASLD, and cardiovascular disorders [110], [111]. Dipeptidyl peptidase 4 (DPP4) inhibition inhibits the N-terminal deactivation of GIP and GLP1, thereby enhancing the natural incretin activity, which is an effective treatment for patients with T2D [112].

Experimental animals and *in vitro* assays demonstrated that flavonoids have an impact on the concentration of GLP-1 as well as the expression and function of DPP4. A study evaluated the effects of pure flavanols and plant extracts enriched in flavanols and procyanidins on GLP-1 levels [113]. An elevation in plasma GLP-1 levels was detected following both acute and long-term ingestion of procyanidins, flavanols, and other monomeric polyphenols [113]. The study conducted by Yang et al. revealed that EGCG effectively reduces fat buildup and alleviates the development of MASLD by inhibiting the expression and function of DPP4 [114]. The hydroxylation at the 4′-position of the B ring, the presence of glucose/rhamnose at the 3- or 7-positions, and the chlorination of flavonoids at the 3- or 8-positions of flavonoids appear to enhance the inhibition of DPP-4. Conversely, the hydroxylation at the 3′ or 3-position, the methoxylation at positions other than 3′ and 4′, and the presence of glucose at the 3′-position appear to have a contrary impact [115]. Although, it should be noted that glycosylation at the 3-position appeared to lessen flavonoids glucosidase inhibition, and opposite to results observed in case of DPP-4 [95], [115].

Flavonoids have a complicated effect on gut hormones in human. The consumption of white rice with a combination of flavonoids in healthy men led to an increase in postprandial plasma GLP-1 levels [116]. Administration of 635 mg of cacao flavonoids 15 min prior to a 50 g oral glucose tolerance test increased the serum GLP-1 level and early insulin levels in 48 healthy participants [92]. Ingestion of 2903 mg of flavonoid for 8 weeks resulted in an increase in early insulin production during the OGTT test. However, it did not have any noticeable impact on GLP-1 levels in overweight or obese individuals [117]. The administration of 500 mg of *Lemon verbena* (35 %) and *Hibiscus sabdariffa* (65 %) flavonoid extracts enriched in flavones/flavonols and anthocyanins, respectively, for 8 weeks resulted in an increase in GLP-1 levels without changing in insulin levels in overweight individuals [118].

Some researchers have shown contradictory findings in clinical trials. Consumption of 600 mg of anthocyanins, with a high-carbohydrate meal resulted in comparable reductions in plasma insulin, GIP, and GLP-1 levels in healthy men and postmenopausal women [119]. The study by Wendy et al. revealed that the consumption of 1200 mg apple flavonoids, or a combination of 600 mg apple flavonoids and 600 mg blackcurrant anthocyanins, resulted in a significant decrease in postprandial insulin, C-peptide, and GIP levels following a mixed carbohydrate meal [120]. They further confirmed that consumption of 0.9 g of apple extract rich in phlorizin (a dihydrochalcone) prior to consumption of 75 g meal of starch/sucrose significantly decreased plasma glucose, insulin, C-peptide, and GIP levels in healthy individuals [91]. A study by Perkin et al. found that polymerized flavonoids (100 mg tea extracts, 50 mg caffeine, and 63 mg catechins) reduced insulin levels in healthy adults who consumed 40 g of fat [107]. Administration of 325 mg of *Lippia citriodora* and 125 mg of *Hibiscus sabdariffa* extracts (rich in anthocyanins) for 60 days markedly decreased GLP-1 levels, but did not affect insulin or peptide YY (PYY) levels [121]. Administration of 615 mg of acute catechin-rich green tea resulted in a significant reduction in GIP levels, but did not affect GLP-1 levels in healthy young men [122].

The concentrations of GLP-1, GIP, and PYY, as well as the felt appetite, remained mostly unchanged when healthy adults consumed 140 g of blueberries (rich in anthocyanins) alongside a higher-carbohydrate breakfast [123]. According to Yanagimoto et al., the consumption of 540 mg green tea catechin and coffee chlorogenic acid (270 mg and 300 mg) resulted in a notable increase in GLP-1 levels, concurrent with a decrease in GIP levels following a high-fat and high-carbohydrate cookie lunch with 75 g of glucose in healthy men [124]. A diverse range of nutrients control GLP-1 and GIP, and their degradation has a relatively brief half-life (GLP-1_1/2_, 1–2 min; GIP_1/2_, 5–7 min) [110], [125]. The digestion and absorption of carbohydrates and fats can be influenced by flavonoids, which in turn stimulate the release of GLP-1 and GIP. Additionally, flavonoids suppress DPP4 function, thereby impeding the breakdown of GLP-1 and GIP and instead promoting their accumulation. However, the precise quantification of GLP-1 and GIP secretion poses significant challenges due to its very low content [125]. Furthermore, the inconsistencies in the inhibitory effects of various flavonoid structures on associated enzymes suggest more detailed studies using single structures for comparison. Collectively, these factors may significantly influence the regulation of GIP/GLP-1 secretion in the human body.

### Flavonoids effect on gut microbiota diversity

#### Gut microbiota and human health

Increasing evidence in the past two decades points to that the intestinal microbiota may significantly influence the metabolic health of the human host and, when aberrant, to the pathogenesis of various common metabolic disorders [126]. Variations associated with obesity have been found in the composition of microorganisms, including *Actinobacteria*, *Clostridium innocuum*, *Eubacterium dolichum*, *Catenibacterium mitsuokai*, *Lactobacillus reuteri*, and *Lactobacillus sakei*, as well as in a few rarer *Archaea species*, like *Methanobrevibacter smithii* [127], [128]. Undigested flavonoids, accounting for 90–95 % of the overall flavonoid consumption, accumulate in the large intestine and subsequently alter the composition of gut microbiota [129], playing a role in disease management as one of its action mechanisms.

#### Shaping gut microbiota

Flavonoids improve MetS and other disease models by modulating the gut microbiota or metabolites in animal models (Fig. 3**D**). The oral EGCG intake suppresses colitis and is associated with short chain fatty acids (SCFAs)-producing bacteria *Akkermansia* and SCFAs, as confirmed by fecal microbiota transplantation (FMT) [130]. Additionally, cranberry extract (rich in proanthocyanidins, flavonols, and anthocyanins) enhances metabolic effects by modifying the characteristics of the MetS caused by a high fat/high sucrose diet, linked to a proportional rise in *Akkermansia* abundance [131]. Citrus polymethoxyflavones (PMFs) may reduce MetS by regulating amino acid metabolism through the manipulation of gut microbiota. PMFs significantly increased the abundance of the commensal bacterium *Bacteroides ovatus*, and administration of *B. ovatus* by gavage decreased branched chain amino acids levels and relieved MetS in high-fat diet fed mice [8]. A diet high in isoflavones selectively regulates the production of lipopolysaccharides (LPS) by the gut microbiota, therefore promoting an anti-inflammatory response and reducing the severity of experimental autoimmune encephalomyelitis [132]. Furthermore, apart from the alterations in gut flora, the metabolites of flavonoids may also exhibit protective effects against certain disorders. For instance, supplementing a high fat diet with flavonoids (anthocyanins, 12.5–17.5 %) increased the concentrations of 4-hydroxyphenylacetic acid (4-HPAA) in the portal plasma and reduced obesity. Additionally, consistent administration of 4-HPAA was enough to reverse hepatic steatosis [9]. Protocatechuic acid is the metabolite of cyanidin-3 to O-β-glucoside produced by the gut microbiota and functions as an antiatherogenic agent [133].

Table 4 summarizes flavonoids effect on gut microbiota in clinical trials. A 6-month regimen of 3–4 cups/day of green tea and Mankai aquatic plant (Green-Mediterranean diet) in humans resulted in significant changes in composition, including increased abundance of the genus *Prevotella* and enzymatic functions related to branched-chain amino acids degradation, as well as decreased abundance of the genus *Bifidobacterium* and enzymatic functions relevant to branched-chain amino acid biosynthesis [134]. Administration of *Aronia* flavonoid extract (rich in flavonol and anthocyanins) for 12 weeks resulted in enhanced development of *Anaerostipes* [135], a species known for its metabolic capabilities in inositol breakdown and the conversion of 3-hydroxybutanoyl-CoA to butyrate [136]. Additionally, it has been shown to exert negative correlations with body weight, waist circumference, and BMI [137]. Dietary intake of grape powder (rich in catechin, anthocyanins, flavonoids) showed a notable rise in *Akkermansia*, *Flavonifractor*, and *Lachnospiraceae_UCG-010*, but a decline in *Bifidobacterium* and *Dialister* at the genus level in healthy individuals [138]. In healthy males, consuming de-alcoholized red wine (anthocyanins, flavan-3-ols, and flavonols) at a dosage of 272 mL/day 4 weeks significantly boosted population of *Enterococcus*, *Prevotella*, *Bacteroides*, *Bifidobacterium*, *Bacteroides uniformis*, *Eggerthella lenta*, and *Eubacterium rectale* groups [139]. Consumption of de-alcoholized red wine for 4 weeks at a dosage of 272 mL/d resulted in an increase in the population of fecal *Bifidobacteria* and *Lactobacillus* (bacteria that protect the intestinal barrier), butyrate-producing bacteria (*Faecalibacterium prausnitzii* and *Roseburia*), as well as less desirable bacterial genera such as LPS producers [140]. A notable increase in *B. longum* subsp. *infantis* was detected in the feces of healthy male volunteers following a 6-week treatment with wild blueberry drink, which contains 325 mg anthocyanins [141]. However, the study by Janssens et al. found no notable alterations in gut microbiota among those who consumed green tea (0.56 g/d EGCG + 0.28–0.45 g/d caffeine) over 12 weeks [142]. Similar observation was found in the gut flora of elderly healthy individuals who were administered 302 mg of anthocyanins from blueberries for 12 weeks [143]. Identification of action mechanism behind mitigation of prebiotic action in case of these drugs coadministration should now follow.

**Table 4:** Flavonoids influence gut microbiota.

Flavonoids (Dosages)	Models	Key findings	References
560 mg EGCG + 350 mg caffeine	Normal subjects	12 weeks, no change	[142]
296 mg anthocyanins and 1040 mg flavonoids	Middle aged healthy	4 weeks, no change	[300]
302 mg anthocyanins blue berry	Healthy elder individuals	12 weeks, no change	[143]
116 mg aronia extract (Flavonol and anthocyanins)	Healthy male	12 weeks increased *Anaerostipes*	[135]
272 mL/d red wine flavonoid (Anthocyanins, flavan-3-ols, flavonols)	Healthy male	4 weeks significantly increased *Enterococcus*, *Prevotella*, *Bacteroides*, *Bifidobacterium*, *Bacteroides uniformis*, *Eggerthella lenta*, and *Blautia coccoides–Eubacterium rectale* groups	[139]
375 mg blue berry anthocyanins and 127.5 mg of chlorogenic acid	Healthy male	6 weeks *B. longum* subsp. *infantis*	[141]
272 mL/d De-alcoholized red wine (Anthocyanins, flavan-3-ols, flavonols)	Obese patients	4 weeks increased *Bifidobacteria* and *Lactobacillus* and butyrate-producing bacteria (*Faecalibacterium prausnitzii* and *Roseburia*) and decrased *Escherichia coli* and *Enterobacter cloacae*.	[140]
282 mg/day EGCG + 80 mg/day resveratrol	Overweight men and women	12 weeks EGCG + RES supplementation significantly decreased Bacteroidetes in men but not in women	[301]
46 g grape powder (catechins, proanthocyanidins, anthocyanins)	Healthy adults	4 weeks increased *Akkermansia*, *Flavonifractor* and *Lachnospiraceae_UCG-010*, but a decrease in *Bifidobacterium* and *Dialister*	[138]
600 mg flavonoids in orange juice (Hesperetin, apigenin, naringenin)	Young adults with depressive symptom	The abundance of the *Lachnospiraceae* family (*Lachnospiraceae_uc*, *Eubacterium_g4*, *Roseburia_uc*, *Coprococcus_g2_uc*, *Agathobacter_uc*) increased	[302]
320 mg/day pomegranate extract (ellagitannin-rich)	MetS patients	4 weeks increased *Lactococcus*, *Bifidobacterium*, *Hespellia*, *Aestuariispira*, and *Tessaracoccus*, and decreased *Bacillaceae*, *ClostridiumXIVa*, *Weisella*	[303]
1391 mg polyphenol-rich diet	Older subjects (age > 60)	8 weeks significant increase in fiber-fermenting and butyrate-producing bacteria such as the family *Ruminococcaceae* and members of the genus *Faecalibacterium*, decrease in *Streptococcus* spp.	[276]
30 g/day cranberry powder (proanthocyanidins, anthocyanins)	Healthy adults	An increase in the Bacteroidales, Lachnospira and Anaerostipes, a decrease in the Oribacterium	[304]

FMT shows great potential in the management of body weight and MetS. A study revealed that a diet rich in flavonoids caused a notable alteration in the composition of the microbiome in overweight individuals over the period of weight loss. Following FMT, the modifications in the gut microbiota caused by the diet rich in flavonoids enhance the retention of particular bacteria related to weight reduction, such as *Alistipes putredinis* and *Bacteroides vulgatus*. This, in turn, reduces weight gain, waist circumference increase, and insulin rebound [144]. While the ability of flavonoids to mitigate MetS through gut microbiota in animal models is well-documented, there is still a scarcity of research in the general population. This gap may be attributed to the greater vulnerability of the gut flora of the participant group in clinical trials to external environmental factors, such as diet and medications, in clinical trial participants.

### Flavonoids suppress gastrointestinal inflammation resulting from aberrant metabolites by the gut microbiota

A robust intestinal barrier prevents the excessive translocation of LPS into the circulatory system. Diet, stress, infections, and dysbiosis can undermine the integrity of the gut barrier, resulting in elevated levels of LPS. In obese participants, consumption of 272 mL of dealcoholized red wine (anthocyanins, flavan-3-ols, and flavonols) for 30 days resulted in a significant 60 % reduction in LPS levels [140]. In contrast, a similar consumption of 272 mL of dealcoholized red wine for 20 days did not result in any significant differences in the change of LPS among middle-aged healthy individuals, however, it did increase the levels of *Bifidobacterium* and *Prevotella*, which may indirectly exert beneficial effects by reducing LPS levels [145]. The administration of cranberry extract (proanthocyanidins 76 mg /day) for two months did not effectively restore the levels of plasma LPS and uremic toxin generated by the gut microbiota in individuals diagnosed with chronic kidney disease [146].

The gut microbiota enzymatically processes choline and carnitine to liberate trimethylamine, which is subsequently transformed into trimethylamine N-oxide (TMAO). An elevated concentration of TMAO is linked to a higher likelihood of developing atherosclerosis, myocardial infarction, stroke, and other cardiovascular diseases. Administration of 600 mg of grape pomace flavonoid extract for 4 weeks resulted in a significant 63.6 % reduction in TMAO levels in healthy individuals [147]. Additionally, administration of 300 mg of tomato extracts high in flavonoids (8–10 % flavonoid derivatives) for 4 weeks can reduce plasma TMAO levels in overweight and obese individuals [148]. Nevertheless, immediate administration of flavanol did not decrease fasting TMAO levels in individuals with increased circulating TMAO [149]. Similarly, 2903 mg of mixed flavonoids for 8 weeks did not impact TMAO levels in humans at high-cardiometabolic risk [150].

Daily administration of 150 mg/day dose of French maritime pine bark extract (67–75 % procyanidins, 4–10 % catechin) for 12 weeks resulted in significant enhancement in plasma total thiol content, total antioxidant capacity (TAC), plasma activity of manganese superoxide dismutase (MnSOD) and catalase, as well as elevated the transcript level of nuclear factor erythroid 2-related factor 2 (Nrf2), MnSOD, and catalase in peripheral blood mononuclear cells (PBMCs) of postmenopausal women with osteopenia [151]. Daily grape extract for one year resulted in a notable decrease in serum alkaline phosphatase (ALP) and interleukin-6 (IL-6) levels. Additionally, the levels of pro-inflammatory cytokines CC-chemokine ligand 3 (CCL3), interleukin-1β (IL-1β), and tumor necrosis factor α (TNF-α) were significantly reduced in PBMCs of hypertensive male patients with T2D [152]. Administering a high-fat meal challenge including cocoa rich in flavonoids (20 g cocoa powder containing 960 mg polyphenols, 480 mg flavanols, 201 mg proanthocyanidins, and 40 mg epicatechin) reduced serum interleukin-18 levels in persons with T2D [153]. Furthermore, administration of 250 mg of pomegranate peel extract (rich in flavonoids and tannins) for 8 weeks resulted in a significant decrease in inflammatory markers (IL-6, TNF-α, high sensitivity C reactive protein), oxidative stress indicators (thiobarbituric acid reactive substances, NO_2_–, O_2_–), and homocysteine levels, while the serum TAC exhibited an increase [154]. Collectively, these findings confirm that flavonoids have advantageous effects in decreasing LPS, TMAO, and pro-inflammatory cytokines, which are also closely associated with gut microbiota. A more comprehensive investigation of clinical data is required.

## Health benefits of flavonoids

MetS, which includes overweight/obesity, hyperglycemia, hypertension, and hyperlipidemias, increases the likelihood of developing cardiovascular diseases, T2D, MASLD, and cancer [155]. The National Institutes of Health database mostly includes flavonoid clinical trials that specifically target MetS [20]. Upon absorption, flavonoids initially exert their effects on endothelial cells, then traverse the portal vein, into the liver, and thereafter circulate throughout the body. In this section, we have consolidated the existing information regarding the role of flavonoids implicated in endothelial dysfunction, MASLD, cardiovascular diseases, obesity, hyperlipidemia, T2D, and cancer, aiming to elucidate the possible involvement of flavonoids in these significant disorders. Clinically, it is challenging to completely differentiate between these conditions. For instance, obese people often present with overlapping conditions such as MASLD, hyperlipidemia, and hypertension. Therefore, we have made a concerted effort to focus on these diseases during our literature search.

### Flavonoids alleviate endothelial dysfunction

#### Regulation of endothelial function

The endothelium is a substantial organ that has a crucial function in maintaining the efficient blood circulation throughout the body. The vascular endothelium regulates the constriction (vasoconstriction) and dilation (vasodilation) of your blood vessels. Endothelium damage confers a susceptibility to various health complications such as atherosclerosis and associated cardiovascular disorders [156]. Atherosclerosis advances through a prolonged preclinical phase without noticeable symptoms, and ultimately presents clinically, often in middle age. Under normal conditions, endothelial nitric oxide synthase (eNOS) in endothelial cells catalyzes nitric oxide (NO) production in the lumen of blood vessels, which hinders platelet aggregation and thrombosis [157]. Paracrine nitric oxide (NO) stimulates soluble guanylate cyclase (sGC) to generate cyclic guanosine monophosphate (cGMP), which activates protein kinase g (PKG) in smooth muscle cells (SMCs). The activation of sarcoplasmic/endoplasmic reticulum calcium-ATPase (SERCA) and the excretion of intracellular Ca^2+^ by the plasma membrane calcium-transporting ATPase (PMCA) are mechanisms by which cGMP and PKG contribute to the depression of intracellular Ca^2+^ levels [158]. Moreover, PKG stimulates the outflow of K^+^ ions by the large-conductance Ca^2+^-sensitive potassium channel (BK_Ca_), which increases the extracellular potential of the cell, therefore decreasing the influx of Ca^2+^ ions through the L-type calcium channel (LTCC). Finally, PKG facilitates the process of dephosphorylating the myosin light chain by means of the corresponding phosphatase [159]. Collectively, these alterations facilitate vasorelaxation. Under normal conditions, perivascular adipose tissue influences this phenomenon by the release of NO and adiponectin, which stimulates the NO synthesis in SMCs [160], [161]

Liver sinusoidal endothelial cells (LSECs) normally regulate the quiescence of hepatic stellate cells (HSCs) by a NO-dependent mechanism. Under physiological shear stress, LSECs trigger the activation of the transcriptional factor KLF2, resulting in the secretion of vasodilating agents such as NO and a reduction of vasoconstrictive molecules like endothelin-1 [162]. In MASLD or cirrhotic liver, LSECs undergo capillarization, resulting in the loss of their fenestrae and the appearance of a basement membrane [163]. Capillarized LSECs promote the activation of hepatic stellate cells, stimulate steatosis, and consequently drive collagen deposition and fibrosis [164]. This alteration is linked to endothelial dysfunction, which means that higher shear stress no longer causes vasodilation but rather vasoconstriction, resulting in elevated intrahepatic resistance. Simvastatin recapitulates the protective influence of KLF2 and enhances the phenotype of HSC by activating a NO-dependent pathway [165], [166].

#### Regulation of endothelial function by flavonoids

**Fig. 4:**
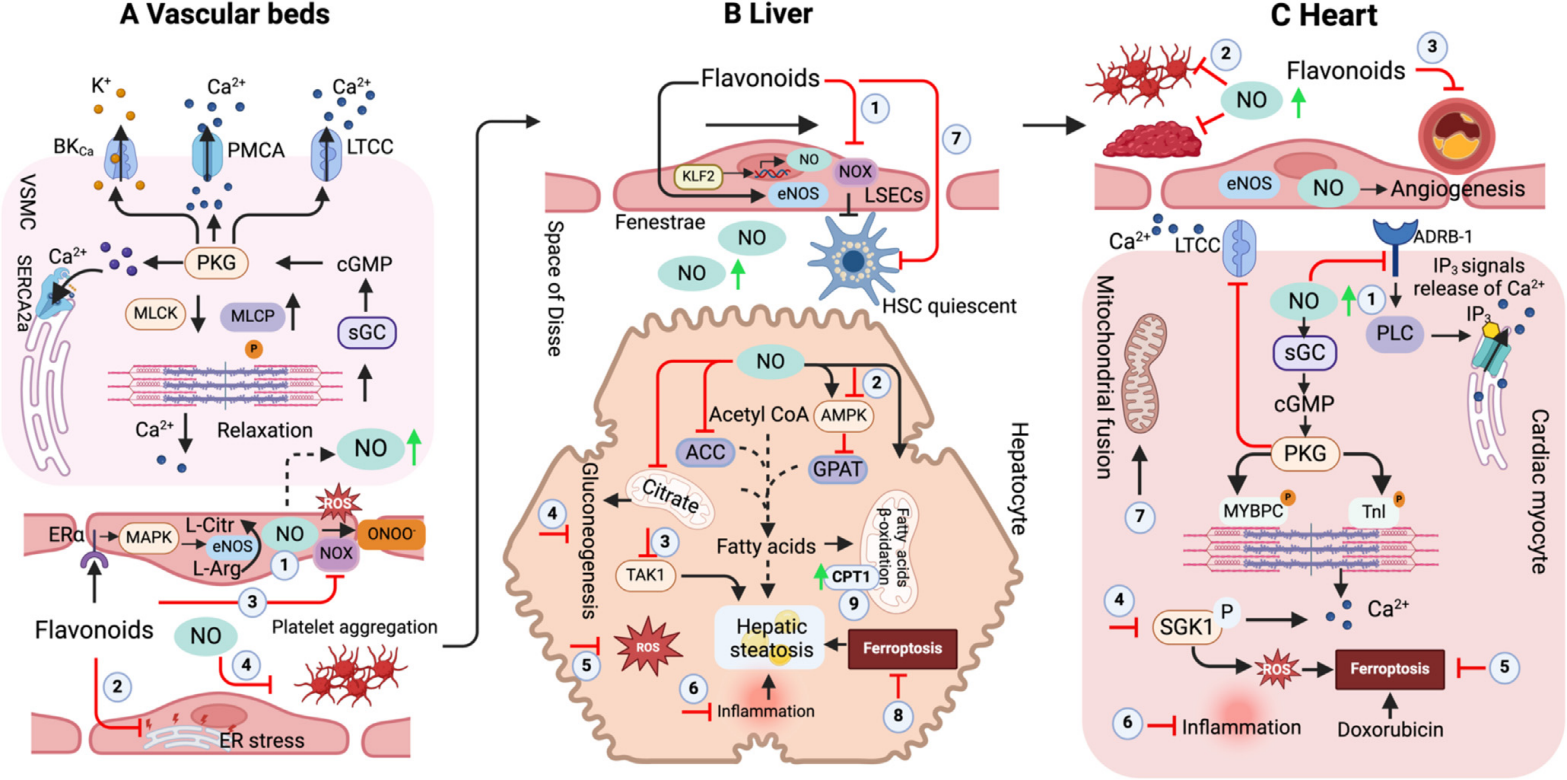
**The roles of flavonoids in vascular, liver, and heart. A. Vascular beds.** Dietary flavonoids demonstrate increased NO release (1), inhibited ER stress (2), NOX activity (3), and platelet aggregation (4), improving the endothelial function. **B, Liver.** Dietary flavonoids alleviate hepatic lipid accumulation by activation of AMPK and TAK1(2–3), reducing ROS and inflammation (5–6), inhibition of HSC activation (7), suppressing ferroptosis (8), and activating hepatic CPT1. **C, Hearts.** Dietary flavonoids increase NO synthesis (1), inhibit platelet aggregation (2) as well as improve lipid metabolism (3), alleviating atherosclerosis. In addition, inhibition of SGK1(4), ferroptosis (5), inflammation (6) and mitochondrial fusion (7) to improve cardiovascular disease. ACC, acetyl CoA carboxylase; AMPK, AMP-activated protein kinase; BK_Ca_, large-conductance Ca^2+^-sensitive potassium channel; CPT1, carnitine palmitoyltransferase 1; eNOS, endothelial nitric oxide synthase; ER, endoplasmic reticulum; GPAT, glycerol-3-phosphate acyltransferase; HSC, hepatic stellate cells; L-arg, L-arginine; L-citr, L-citrulline; LSECs, liver sinusoidal endothelial cells; LTCC, L-type calcium channel; MAPK, mitogen-activated protein kinase; MLCP, myosin light chain phosphatase; MYBPC, myosin-binding protein C; PLC, protein lipase C; NO, nitric oxide; NOX, NADPH oxidase; PKG, protein kinase G; PMCA, plasma membrane calcium-transporting ATPase; SERCA, sarcoplasmic/endoplasmic reticulum calcium-ATPase; SR, sarcoplasmic reticulum; sGC, soluble guanylate cyclase; SGK1, Serum/glucocorticoid regulated kinase 1; TAK1, TGF-β-activated kinase 1; TnI, troponin I; VSMCs, vascular smooth muscle cells.

Flavonoids regulate endothelial function through various mechanisms. For instance, procyanidin B2 reduced vascular endothelial stress in a mouse model via the activation of peroxisome proliferator-activated receptor δ (PPARδ) [167]. Hesperidin and naringin enhance eNOS expression in human vein endothelial cells through the mitogen-activated protein kinase (MAPK) signaling pathway [168]. The long-term treatment of epicatechin protects against hypertension by reducing oxidative stress and decreasing NADPH oxidase (NOX) activity [169]. Several flavonoids, such as quercetin, hesperetin, and daidzein, also enhance the activity of eNOS, decrease oxidative stress, stimulate vasodilation by regulating K^+^ and Ca^2+^ ion channels, and so augment endothelial function [170]. The study by Zhao et al. demonstrates that vitexin effectively suppresses flow-induced endothelial inflammation by inhibiting apurinic/apyrimidinic endonuclease1 [171] (Fig. 4 **A**).

Numerous human studies have demonstrated the protective and functional benefits of flavonoids for endothelial cells. The administration of cocoa flavanols at a dosage of 1350 mg enhances endothelial function in both the brachial artery and the femoral artery in both healthy individuals and those with T2D [172]. Additionally, consuming of 30 mg of flavonoids from dark chocolate for 18 weeks, with minimal habitual cocoa intake, effectively decreased blood pressure and enhanced the production of vasodilative NO [173]. Similarly, administration of 302 mg of anthocyanins from blueberries for 12 weeks improved vascular function in healthy older persons, leading to a notable rise in flow-mediated dilation (FMD) and a decrease in 24-hour ambulatory systolic blood pressure measures [143]. Consumption of Montmorency tart cherry juice (73.5 mg anthocyanin) immediately lowers SBP in men with early hypertension [174]. Short-term (2 h post-consumption of a chocolate bars) and long-term (4 weeks, two chocolate bars daily) improvement in vascular function through platelet function suppression were observed in patients with congestive heart failure (CHF) [175]. Daily administration of 30 mL/day of red wine (dealcoholized) for 4 weeks reduces both systolic and diastolic blood pressure, associated with increased plasma NO levels [176].

In contrast, a study by Corban et al. reported that flavonoid-rich chardonnay seed extracts (0.53 g catechin, epicatechin, and their oligomers procyanidins for 4 months) did not provide any additional benefits compared to flavonoid-free supplements in improving peripheral endothelial function in individuals with peripheral endothelial dysfunction [177]. According to Curtis et al., the consumption of blueberry extracts containing 364 mg anthocyanin and 879 mg phenolics, together with a diet of 64.5 g fat, 84.5 g carbohydrate, and 17.85 g protein, did not have any significant impact on FMD and blood pressure in individuals with MetS (BMI = 31.4) [108]. Furthermore, participants with severe obstructive sleep apnea and no obvious cardiovascular disease showed no improvement in endothelial function after receiving a daily dosage of 300 mg/day of grape juice flavonoids for one month [178]. For 2 weeks, cocoa administration (900 mg flavanols) enhanced insulin-stimulated alterations in the diameter of the brachial artery. However, the effects of cocoa therapy on blood pressure were not statistically significant, demonstrating two weeks of daily ingestion of cocoa high in flavanols is insufficient to lower blood pressure [179]. The variations in the effects on endothelial cells may be attributed to the participants’ health condition as well as the specific type and dosage of flavonoids used.

### Flavonoids improve MASLD

MASLD affects approximately 25–30 % of the global population, making it as one of the most prevalent chronic liver disorders. The increasing incidence of MASLD is partly attributed to the rising obesity rates [180]. MetS, encompassing disorders such as obesity, insulin resistance, hypertension, and dyslipidemia, is the primary cause of MASLD [181]. On 14th March 2024, the US Food and Drug Administration (FDA) granted approval to the first medication, resmetirom (an oral, liver-directed, thyroid hormone receptor beta–selective agonist) for metabolic dysfunction-associated steatohepatitis (MASH) [182]. The high incidence and severe consequences of MASLD have generated significant interest and rigorous research to the underlying pathophysiological processes to develop innovative treatments.

Flavonoids exert their influence on liver lipid metabolism through various mechanisms in animal or cell models (Fig. 4 **B**). For example, breviscapine inhibits TGF-β-activated kinase 1 (TAK1) signaling, effectively halting the course of metabolic stress-induced MASH [183]. Hyposide enhances the activation of hepatic farnesoid X receptor (FXR) by increasing conjugated bile acids in the rat liver, promoting cholesterol efflux and bile acid excretion from hepatocytes, ultimately reducing lipid accumulation [184]. In Mcpip^fl/fl^ LysM^Cre^ mice, which resemble symptoms of liver inflammation reported in humans, diosmetin effectively decreased LSEC activation and liver inflammation, suggesting potential for novel anti-inflammatory treatments for the liver [185]. Quercetin mitigates liver fibrosis by modulating glycolysis in LSEC [186]. In high fat induced animal models, citrus flavonoids (nobiletin and naringenin) significantly inhibit liver TG accumulation by decreasing hepatic lipid synthesis and increasing hepatic FA-oxidation [187], [188], [189]. Silybin has been shown to have anti-inflammatory and anti-fibrogenic properties in human hepatic stellate cells [190]. Low dose of EGCG suppresses gluconeogenesis in primary hepatocytes [191]. Diet-induced hepatic steatosis was improved by long-term baicalin administration. This flavonoid directly activated hepatic carnitine palmitoyltransferase 1 (CPT1) with isoform selectivity, which sped up the inflow of lipids into mitochondria for oxidation [192]. Zhang et al. demonstrated that troxerutin (a trihydroxyethylated derivative of the natural bioflavonoid rutin) mitigates oxidative stress, therefore providing protection against liver inflammation induced by 2,2,4,4-tetrabromodiphenyl ether [193]. Anthocyanins possess potential as candidate therapeutics for MASLD by targeting AMP-activated protein kinase (AMPK) [194]. Various flavonoids alleviate MASLD through multi-targets. Although flavonoids can improve endothelial cell dysfunction, there is no evidence indicating that the amelioration of MASLD by flavonoids is dependent upon the enhancement of endothelial cell function.

Several positive results have emerged from clinical studies investigating the use of dietary flavonoids in the treatment of MASLD in human. A study conducted by Meir et al. found that following a Green-Mediterranean diet including green tea (3–4 cups/day) and Mankai green smoothie (1240  mg/day total flavonoids) for 18 months, the prevalence of MASLD decreased to 31.5 % compared to 54.8 % in healthy dietary guidelines [195]. Administration of 200 mg of litchi-derived flavonoids for 24 weeks reduced hepatic steatosis in patients with MASLD (grade ≥ 2) [196]. Supplementation of 1 g hesperidin capsule for 12 weeks significantly reduced alanine aminotransferase (ALT), γ‐glutamyltransferase, total cholesterol, triglyceride, and hepatic steatosis in MASLD patients [197]. Consuming 40 g of dark chocolate (containing more than 85 % cocoa) daily for 2 weeks decreased the levels of soluble NADPH oxidase isoform 2 (NOX2)-derived peptide, which serves as a marker of NOX2 activation, as well as serum cytokeratin-18 and ALT, with antioxidant activity achieved by down-regulating NOX2 in patients with MASH [198]. A 3 times daily administration of 900 mg of *Hibiscus sabdariffa* extract enriched in anthocyanins decreased body weight, abdominal fat, serum free fatty acids, and improved hepatic steatosis in individuals with a BMI ≥ 27[199]. In contrast, administration of 700 mg of silymarin three times daily for 48 weeks did not result in a 30 % or more reduction in MASLD activity scores. However, it was shown to be linked with a much better improvement in fibrosis compared to a placebo group. Additionally, the treatment was considered safe and well tolerated [200]. Further research is needed to validate these findings in a larger trial and extended through longer time.

### Flavonoids alleviate cardiovascular disease

The beneficial effects of flavonoids on cardiovascular diseases have been demonstrated in numerous disease models [201], [202], [203]. In a rat model of autoimmune myocarditis, tea catechins have been shown to enhance left ventricular function, reduce myocardial inflammation and fibrosis, and modify cytokine expression [204]. Herbacetin, a novel inhibitor of serum/glucocorticoid regulated kinase 1 (SGK1), effectively inhibits cardiomyocyte hypertrophy both *in vitro* and *in vivo*. It also decreased the generation of reactive oxygen species (ROS) and the buildup of calcium [202]. Nanofibers impregnated with baicalin efficiently suppressed ferroptosis and reduced the cardiotoxicity caused by doxorubicin [203]. Additionally, the compound breviscapine inhibits pathological cardiac hypertrophy by specifically targeting the FOXO3a-mitofusin-1 mediated mitochondria fusion [205]. Furthermore, flavonoids exhibit potential protective effects against cardiovascular diseases through anti-fibrotic, anti-inflammatory, and antiplatelet mechanisms [206] (Fig. 4 **C**).

Consuming flavonoids can help prevent cardiovascular problems in the general population. In adults at high cardiovascular risk, flavonoids consumption was associated with a 37 % relative decrease in all-cause mortality [18]. Incorporating 3–4 cups of green tea and Mankai aquatic plant into a Green-Mediterranean diet for 6 months resulted in improved cardiometabolic health, evidenced by changes in specific biomarkers such as body weight, waist circumference, blood pressure, and glycemic profile, particularly in individuals with abdominal obesity/dyslipidemia [134]. The Dietary Approaches to Stop Hypertension (DASH) diet, characterized by a high intake of fruits, vegetables, and low-fat dairy products, together with a reduction in saturated fat and cholesterol, effectively lowers blood pressure [207]. Tea flavonoid supplementation at a dosage of 200 mg/100 mg was shown to enhance the elimination of N-acetyl-S-(2-carbamoyl-2-hydroxyethyl)-L-cysteine, a crucial mercapturic acid produced during the glycidamide metabolism in the urine. This suggests that tea flavonoids may help prevent cardiometabolic toxicity caused by acrylamide [208]. In individuals with coronary artery disease, both short-term (2 h) and long-term (4 weeks) black tea consumption enhanced endothelium-dependent flow-mediated dilation of the brachial artery [209]. In middle-aged unmedicated individuals with cardiovascular risk factors, consuming berries (mainly, 515 mg anthocyanins and 115 mg procyanidins) for 8 weeks effectively suppressed platelet activity, increased high density lipoprotein (HDL)-cholesterol levels, and reduced systolic blood pressure [210]. Dietary ingestion of 50 g freeze-dried blueberries (rich in anthocyanins) for 8 weeks reduces cardiovascular risk factors in obese men and women with MetS by lowering blood pressure, plasma oxidized LDL, and serum malondialdehyde and hydroxynonenal levels [211]. Following a 6-week regimen of 136 mg of oleuropein and 6 mg of hydroxytyrosol daily, pre-hypertensive individuals showed significant reductions in systolic blood pressure, diastolic blood pressure, total cholesterol, LDL cholesterol, and triglycerides [212].

However, according to Kirch et al., the daily consumption of 25 mg of pure (−)-epicatechin for 2 weeks does not decrease cardiometabolic risk factors in overweight-to-obese individuals. Therefore, the cardioprotective effects of regular cocoa consumption, which are attributed solely to (−)-epicatechin, should be reevaluated [213]. Administration of 105.9 mg of aronia flavonoid (procyanidins, cyanidin-3-galactoside et al.) for 12 weeks resulted in improvements in arterial parameters such as augmentation index and pulse wave velocity. However, there were no significant changes in blood pressure, endothelial function, or blood lipid levels [214]. Prior studies have shown that flavonoids exhibited potential in the treatment of cardiovascular disease (Table 5). However, identifying specific dietary flavonoids or combinations of flavonoids that can effectively address cardiovascular disease remains a challenge in both animal models and human populations.

**Table 5:** Flavonoids and cardiovascular disease.

Flavonoids	Participants	Test meal	Cardiovascular disease	
Apple flavanol extractapple PCs extract	43 healthy subjects with moderately elevated blood pressure	70 mg monomeric flavanols, 65 mg PCs for 28 days	No significant effects on BP, NO, LDL, glucose [305]	
Flavonoid‑rich chardonnay seed	89 patients with PED	530 mg 12 weeks	No incremental benefits for PED [177]	
Grape juice flavonoids	40 patients with OSA	300 mg/day 1 month	No incremental benefits in RHI and blood pressure [178]	
Flavonoid-rich dark chocolate	44 older subjects with prehypertension or stage 1 hypertension	30 mg 18 weeks	Reduced BP and improved bioactive nitric oxide [173]	
Anthocyanins and PCs	72 middle-aged unmedicated subjects with cardiovascular risk factors	150 g 8 weeks	Reduced BP, inhibited platelet function, and elevated HDL-cholesterol concentration [210]	
Anthocyanins	66 obese men and women	50 g dried blue berries 8 weeks	Reduced BP, oxidized LDL, serum MDA, and HNE [211]	
Flavonol and anthocyanins	66 healthy men	12 mg aronia whole fruit or 116 mg aronia extract 12 weeks	Improved endothelial function [135]	
Cocoa flavanol	11 healthy and 11 T2D	1350 mg 2 h	Increase endothelial function both in BA and FA [172]	
Montmorency tart cherryAnthocyanins	15 men patients with early hypertension	178.45 mg phenolic 3 h	Lower systolic blood pressure [174]	
Flavanol-rich chocolate	20 patients with CHF	2 h after ingestion of a chocolate bar and 4 weeks two chocolate bars/day	Improves vascular function [175]	

BP, blood pressure; MDA, malondialdehyde; HNE, hydroxynonenal; BA, brachial artery; FA, femoral artery；PED, peripheral endothelial function; OSA,obstructive sleep apnea syndrome; RHI, reactive hyperemia index; PCs, procyanidins; T2D, type 2 diabetes; CHF, congestive heart failure.

### Flavonoids reduced overweight/obesity

China has one of the highest obesity prevalence rates globally, posing a significant challenge to the nation's healthcare system [215]. Current recommendations for the therapy of obesity lack sufficient support from clinical trials conducted in Chinese populations. The implementation of effective lifestyle interventions is crucial for the management of obesity. Multiple studies have consistently shown that the consumption of dietary flavonoids is associated with a decreased risk of obesity.

**Fig. 5:**
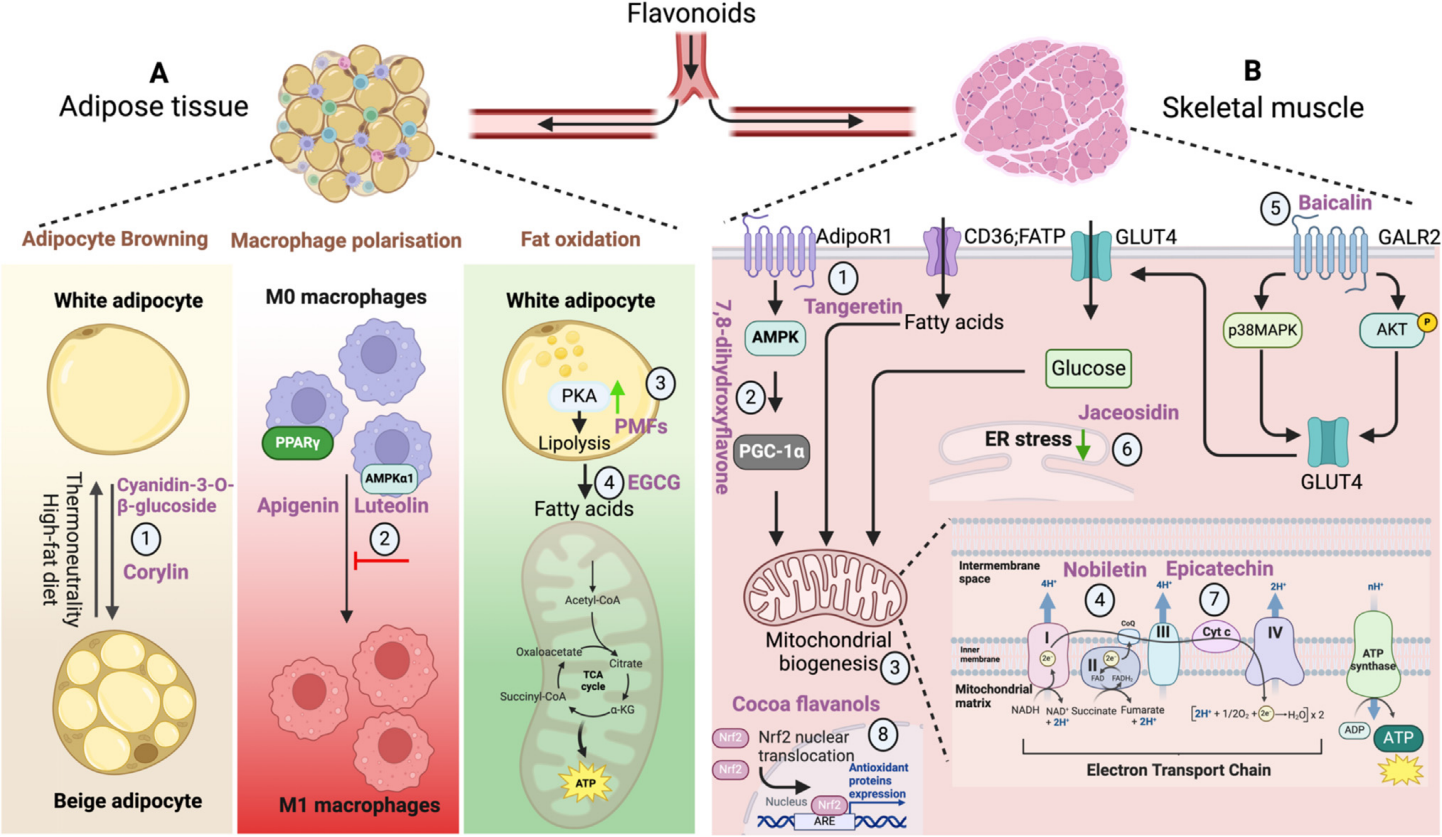
**The roles of flavonoids in adipose tissue (A) and skeletal muscle (B).** A, Dietary flavonoids promotes adipose tissue browning (1), activates AMPKα1/PPARγ in adipose tissue macrophages to inhibit inflammatory polarization (2), increases lipolysis through activation of PKA downstream signaling (3), and fatty acids oxidation (4). B, Dietary flavonoids target AdipoR1 to improve aerobic metabolism and muscle endurance (1), activate AMPK/PGC-1α pathways to enhance mitochondrial biogenesis (2–3), strengthen mitochondrial respiratory chain complexes architecture and activity (4), promote AKT and p38MAPK pathways to translocate GLUT4 (5), improve endoplasmic reticulum stress (6), enhance mitochondrial COX activity (7), and activate Nrf2, raising antioxidant protein levels (8). AdipoR1, adiponectin receptor 1; AMPKα1, AMP-activated protein kinase alpha 1; COX, cytochrome *c* oxidase; ER, endoplasmic reticulum; FATP, fatty acid transporter protein; GALR2, galanin receptor 2; GLUT4, glucose transporter 4; LPS, lipopolysaccharide; MAPK, mitogen-activated protein kinases; Nrf2, nuclear factor erythroid 2-related factor 2; PGC-1α, peroxisome proliferator-activated receptor-gamma coactivator 1α; PKA, protein kinase A; PPARγ, peroxisome proliferator-activated receptor γ; PMFs, polymethoxyflavones.

Flavonoids affecting adipose tissue tend to be multi-targeted in mouse models (Fig. 5**A**). Luteolin reduced the harmful consequences of diet-induced obesity and associated comorbidities through the interactions between adipose and hepatic tissue,[216]. Corylin, a flavonoid compound extract from *Psoralea corylifolia L*., promoted lipid metabolism, activates brown adipose tissue (BAT), and browns white adipocytes to produce anti-obesity actions [217]. Cyanidin-3-O-β-glucoside reversed the high fat cholesterol diet caused disruptions to the activation of BAT and the expression of adipokines in BAT [218]. Dietary luteolin reduced adipose tissue macrophage inflammation and improved insulin resistance in diet-induced obese mice via the stimulation of AMPKα1 signaling in adipose tissue macrophages [219], [220]. Apigenin, a natural flavonoid, reduces obesity-related inflammation by controlling macrophage polarization after PPARγ activation [221]. After being treated with PMFs, adipocytes' mitochondrial content and oxidative metabolism rose noticeably. Additional, treatment with PMFs boosted lipolysis by triggering downstream signaling of protein kinase A (PKA). Adipocyte-specific adipose triglyceride lipase deletion mice did not exhibit the anti-obesity effects, suggesting that cytosolic lipase-dependent pathways underlie the PMFs action [222].

The Green-Mediterranean diet, which is twice higher in dietary flavonoids (green tea 3–4 cups/day + 800 mg/day flavonoids) and lower in red and processed meat, showed a substantial reduction in visceral adipose tissue (VAT) over a period of 18 months. This suggests that a flavonoid-enriched diet may be effective in targeting VAT [223]. A low-fat plant-based dietary regimen decreases body weight by lowering energy consumption and enhancing postprandial metabolism. The alterations are linked to decreases in hepatocellular and intramyocellular fat, along with enhanced insulin sensitivity [224]. The DASH diet can effectively mitigate many metabolic risks in both genders, decreasing body weight and triglycerides level [225]. The administration of 400 mg/day of decaffeinated green tea flavonoids for 12 weeks effectively reduced the proportion of body fat in girls aged 6–10 who were obese [226]. Administration of PMFs purified from *K. parviflora* reduced visceral fat in Japanese overweight adults [227]. Additionally, consumption of a green tea extract containing 583 mg of catechins for 12 weeks resulted in a decrease in body fat, SBP, and LDL cholesterol levels in visceral fat-type obese individuals [228], This effect is linked to the activation of lipid catabolism in the liver [229]. When consumed orally, tea catechin and caffeine together produced an immediate increase in energy expenditure linked to an increase in BAT activity as well as a chronic elevation in nonshivering cold-induced thermogenesis in humans, most likely due to the recruitment of BAT [230]. Supplementation with epigallocatechin-3-gallate and resveratrol (EGCG + RES, 282 + 80  mg/day) for 12 weeks enhanced fat oxidation involving the entire body [231]. Supplementation with a combination of EGCG (282 mg/day) and RES (200 mg/day) for 3 days greatly enhanced fasting and postprandial energy expenditure in overweight individuals [232].

However, administration of 25 mg of pure (−)-epicatechin for 2 weeks did not have a significant impact on body weight, fat mass, fat distribution, and other risk factors related to cardiovascular and metabolic health in overweight or obese individuals [213]. Long-term administration of EGCG and RES at doses of 282 mg and 80 mg/day, respectively, for 12 weeks enhanced mitochondrial capacity in permeabilized muscle fibers and promoted fat oxidation compared to a placebo. However, this did not result in improved tissue-specific insulin sensitivity in overweight and obese individuals. Additionally, no changes were seen in the fasting plasma metabolic profile and whole-body fat mass [233]. Numerous ongoing clinical trials are currently enrolling participants to investigate the impact of flavanol-rich chocolate, cranberry extract, cocoa flavonoids, coffee, and tea on weight management and health indicators.

### Flavonoids effect on muscle dysfunction

Muscle affects glucose homeostasis, redox balance, and the body's overall energy metabolism, all of which are critical to metabolic health [234]. Extracellular vesicles and myokines released by skeletal muscle affect other tissues [235]. A number of MetS, such as diabetes and cardiovascular disease, are associated with dysfunctional skeletal muscle [236], [237]. Techniques to improve or preserve the skeletal muscle health can lead to better results and a lower burden of disease. It has long been known that exercise is a cost-effective and efficient form of therapy [238].

In skeletal muscle, dietary flavonoids can affect energy homeostasis in mice (Fig. 5**B**). Tangeretin, a key member of flavonoids in citrus peels, effectively stimulates the expression of genes related to slow oxidative myofibers and enhances the proportion of type I myofibers in mice. This leads to enhanced endurance performance and increased aerobic metabolism. The beneficial impact of tangeretin is facilitated via AdipoR1 and its subsequent AMPK/peroxisome proliferator-activated receptor-gamma coactivator 1α (PGC-1α) pathway [239]. In diet-induced obese mice, 7,8-dihydroxyflavone activated the AMPK/PGC-1α pathways to enhance mitochondrial biogenesis and augment the number of mitochondria in the muscle, leading to systemic energy expenditure and muscular mitochondrial respiration [240]. In aged mice given a HF diet, nobiletin promotes healthy and extensively activates the genes encoding mitochondrial respiratory chain complexes (MRCs) in HF skeletal muscle, strengthening MRC architecture and activity, including complex II activation and supercomplex formation. Together, these pathways result in a dichotomous mitochondrial optimization, which raises ATP synthesis while lowering ROS levels [241]. Insulin resistance and hyperglycemia were reduced with baicalin. In obese mice, the baicalin-induced positive benefits were eliminated by the galanin receptor 2 (GALR2) antagonist M871. In addition, baicalin activated the AKT and p38MAPK pathways in myotubes, resulting in a significant increase in glucose consumption and PGC1α-GLUT4 axis activity [242]. The compound jaceosidin improves endoplasmic reticulum stress and insulin resistance by increasing the expression of sarco/endoplasmic reticulum Ca2 + ATPase 2b (SERCA2b) [243]. Circulating flavonoids influence muscle metabolism through multiple targets.

In peripheral artery disease patients, cocoa flavanols activate Nrf2, raising antioxidant protein levels, preventing skeletal muscle damage, and boosting the quantity of mitochondrial proteins [244]. Administration of epigallocatechin-3-gallate and resveratrol (EGCG + RES, 282 + 80  mg/day) supplements for 12  weeks increased mitochondrial respiration in permeabilized skeletal muscle fibers in males, but did not have the same effect on females. The susceptibility of men to diet-induced, gut microbiota-related enhancements in cardiometabolic health may be higher [231]. Addition of essential amino acids and tea catechins to resistance training enhances skeletal muscle mass in older individuals with sarcopenia, as compared to resistance exercise alone. Notably, the essential amino acid group did not show a significant improvement in muscle gain. However, the combination consumption of essential amino acid and tea catechin resulted in a considerable increase in muscle gain, suggesting catechin benefit muscle gain may due to inhibition of muscle protein breakdown [245]. Amateur cyclists who took 500 mg/day of hesperidin for eight weeks saw a drop in fat mass and an increase in both percentage and total muscle mass [246]. The therapeutic impact of cocoa on walking performance in individuals with peripheral artery disease is evidenced by its ability to enhance mitochondrial cytochrome *c* oxidase (COX) activity, increase capillary density, improve calf muscle perfusion, and decrease central nuclei in calf muscle biopsies [247]. Supplementation with quercetin (1000 mg/day) for 2 weeks helps reduce the degree of muscle injury and strength loss brought on by eccentric exercise because of its potent anti-inflammatory and antioxidant properties [248]. Administration of EGCG (600 mg/day) to individuals with multiple sclerosis for 12 weeks enhances muscle metabolism during moderate activity more significantly in males than in women, maybe due to particular effects on autonomic and endocrine regulation [249]. After being digested, absorbed, and metabolized in the liver, flavonoids themselves or a tiny quantity of metabolites still have an effect on muscle tissue. The mechanism underlying this remains mainly unexplained and has not been satisfactorily explained by the literature. Metabolism of flavonoids may differ between males and females.

### Flavonoids protect against hyperlipidemia

One of its noteworthy characteristics is the way in which flavonoids regulate lipid metabolism. Consumption of 1 cup of blueberries, containing 364 mg of anthocyanin and 879 mg of phenolics, significantly enhanced HDL-C levels and the fractions of HDL particles following a 24-hour high-fat/high-sugar meal [108]. Likewise, administration of 165 mg of mono-glucosyl hesperidin and 387 mg of green tea catechins for 4 weeks markedly reduced fasting blood TG levels in healthy individuals [250]. A dosage of 220 mg of cocoa flavanols for 4 weeks reduced levels of total cholesterol, triglycerides, plasma LDL, and oxidized LDL (oxLDL) in healthy individuals [251]. Additionally, consuming a high-fat meal supplemented with cocoa rich in flavanols (20 g cocoa power) reduced the levels of VLDL and chylomicron particles, while increasing the concentrations of total HDL particles in T2D patients throughout the 6-hour postprandial period [153]. Conversely, the study conducted by Princen et al. did not observe any impact on LDL oxidation following the consumption of green or black tea (6 cups, equivalent to 1.2 g green tea flavonoid) or the intake of a green tea flavonoid isolate (3.6 g of green tea flavonoids per day, equivalent to the consumption of 18 cups of green tea) [252]. Furthermore, as mentioned in the previous sections, dietary intake of flavonoids positively influences lipid digestion and absorption and improves MASLD, potentially benefiting hyperlipidemic patients.

### Flavonoids exhibit antidiabetic action

Higher intakes of anthocyanins, flavan-3-ols, flavonols, flavones, polymers, and proanthocyanidins were linked to a 19 %, 26 %, 28 %, 19 %, 26 %, and 27 % decreased incidence of T2D when comparing high to low flavonoid subclass intakes [253]. Increased dietary consumption of flavonoids is associated with a more beneficial cardiovascular risk factor profile in individuals with T2D, including reductions in LDL-cholesterol, increases in HDL-cholesterol, and improvements in systolic and diastolic blood pressure, HbA1c, and triglycerides levels [254]. In healthy adults, grape pomace flavonoids (1.562 g gallic acid equivalents) decreased postprandial insulin incremental area by 31 % and enhanced insulin sensitivity by 36 % compared to a normal diet [255]. Daily administration of 8 g of dehydrated grape pomace daily for 6 weeks improves insulin sensitivity in individuals at risk for MetS, without impacting other MetS markers [256]. In overweight or obese participants, a high flavonoid diet substantially decreased plasma glucose levels, enhanced early insulin production, and improved post-challenge oral glucose insulin sensitivity [117]. Additionally, a natural blend of grape flavonoids administered at a dosage of 2 g per day for 9 weeks effectively reduced oxidative stress caused by fructose and insulin resistance in the immediate family members of individuals with T2D [257]. Similar results were reported for the antidiabetic action of 240 mL of cranberry juice daily for 12 weeks, together with supplementation of blueberry extract or powder (9.1 to 9.8 mg of anthocyanins, respectively), for 8 to 12 weeks, as manifested by a positive impact on glucose management in individuals with T2D [16]. Contrary to expectations, there is another report showing that administration of 600 mg/day of red wine polyphenols (95 % flavonoids) for 8 weeks did not enhance insulin sensitivity in obese participants [258]. Collectively, dietary flavonoids influence the breakdown and assimilation of carbohydrates, as well as the glucose metabolism in organs such as the liver, thereby promoting the control of glucose balance in the body. Nevertheless, in individuals with significant dysregulation of glucose metabolism, the impact of dietary polyphenol consumption may be limited.

### Flavonoids have evidence to demonstrate efficacy in specific cancer intervention

In 2022, lung cancer was the most prevalent cancer globally, constituting one in eight cancer diagnoses (12.4 % of all cancers), followed by female breast cancer (11.6 %), colorectal cancer (9.6 %), prostate cancer (7.3 %), and stomach cancer (4.9 %) [259]. In China, lung cancer was also the predominant cancer, succeeded by colorectal cancer, thyroid cancer, liver cancer, and stomach cancer, collectively representing 57.42 % of all new cancer cases [260]. While several cell research, animal studies, and preclinical evidence indicate considerable anticancer properties of flavonoids, the clinical results have not been definitive [261], [262].

Drinking tea may help prevent lung and liver cancers. Compared to not drinking tea at all, the consumption of green, black, or unidentified tea has been shown to have a preventive impact in a population database including 19, 433 lung cancer patients and 718, 854 controls. Additionally, drinking tea 2.5 g daily helped prevent lung cancer in people who did not smoke. Lung cancer risk ratios can be further lowered by increasing green tea consumption to 7.5 g daily [263]. A *meta*-analysis has found that tea consumption can prevent the development of primary liver cancer in both males and women, mainly in China and Japan [264]. However, the greatest level of green tea drinking was significantly associated with a lower risk of liver cancer only in women, according to the summary relative risk of nine prospective cohort studies included 465,274 people and 3694 liver cancer cases from China, Japan, and Singapore [265]. Tea intake affects liver cancer and may be related to dose intake. In another meta-analysis, when compared to nondrinkers, the highest green tea consumption (≥5 cups/day) demonstrated a statistically significant correlation with the risk of liver cancer [266]. One or three cups revealed no statistically significant link in analysis of the relationship between the risk of liver cancer [265], [267].

Flavonoids in breast cancer, colorectal cancer, and prostate cancer exhibit heterogeneity or no obvious effect. A meta-analysis found a notable correlation existed between green tea and breast cancer, suggesting that elevated green tea intake may diminish the incidence of breast cancer, especially in long-term, high doses [268]. According to data from case-control studies, women who received the greatest amount of GT had a 19 % lower incidence of breast cancer. However, cohort studies revealed no discernible variation [269], [270]. A comprehensive analysis of 263,311 population-based, prospective cohort studies found green tea intake does not have meaningful impact (aka no preventive or aggravating effect) on the risk of developing gastric cancer [271]. The data from a cohort of 476,160 men and women across 10 European nations showed no significant correlation between total dietary flavonoid intake and the risk of colorectal cancer in either gender [272]. A *meta*-analysis of prospective cohort studies comprising 2,068,137 individuals and 21,437 cases demonstrates that tea drinking does not significantly affect colorectal cancer risk in both genders combined [273]. Administration of 780 mg EGCG for 6 months did not significantly decrease the number of rectal aberrant crypt foci, which are believed to be early indicators of colorectal malignancies, compared to a placebo [274]. In men with biochemically recurring prostate cancer, the administration of both 500 mg and 1000 mg of grape skin extract for 12 months did not result in a substantial extension of the doubling time of prostate specific antigens [275]. Peripheral organ cancer protection appears to be mediated by intricate pathways including flavonoids, their metabolites, or other signaling messengers. The beneficial of tea in cancer prevention has documented in the lung and liver cancer, and the role of other flavonoids species needs to be further elucidated.

### Flavonoids and other diseases

Intestinal permeability has emerged as a new target for disease prevention and therapy. An 8-week regimen of a diet rich in flavonoids (1391 mg/day) enhanced intestinal permeability in older individuals, as measured by serum zonulin levels [276]. A diet providing 724 mg/day of flavonoids from berries, blood orange and juice, pomegranate juice, and green tea for 8 weeks markedly raises serum indole 3-propionic acid levels in older individuals with normal renal function (RF). However, this effect is not seen in individuals with impaired RF [277]. This increase in serum indole 3-propionic acid levels is linked to gut dysbiosis and endotoxin leakage. Investigation of the efficacy of flavonoids in modulating intestinal permeability is an important focus for future study.

Cognitive aging refers to the deterioration of certain cognitive functions that occurs progressively as we age. Over a period of three years, the administration of 500 mg/day of cocoa flavanols improved memory in elderly individuals [278]. A 12-week regimen of 302 mg of anthocyanins from blueberries enhanced cognitive function in healthy older adults [143]. A single dose of 108 mg taxifolin administered to healthy students enhanced their computational skills and reduced mental exhaustion [279]. Regular consumption of 236.5 mL of pomegranate juice (rich in ellagitannin) for 12 months enhanced the capacity to acquire visual knowledge [280]. Other flavonoids, for example fisetin, have tested the efficiency in mild cognitive impairment patients [281]. Nevertheless, the administration of epigallocatechin gallate for 48 weeks, starting with one 400 mg capsule daily for 4 weeks, followed by one capsule twice daily for 4 weeks, and then three capsules daily for 40 weeks, did not alter the course of the disease in individuals with multiple system atrophy [282].

Specific regions of the skin of healthy volunteers were subjected to EGCG, (−)-epicatechin-3-gallate (ECG), (−)-epigallocatechin (EGC), or (−)-epicatechin (EC) treatments. The flavonoid fractions EGCG and ECG showed the most efficacy in suppressing erythema caused by ultraviolet (UV) radiation. In contrast, EGC and EC had minimal impact [283]. A four-week course of EGCG therapy at a dosage of 800 mg/day did not offer any protection against UV-induced erythema [284]. In summary, flavonoids exhibit promising potential in enhancing gut health, cognitive function, and providing protective effects against skin damage, although further research is necessary to establish their efficacy across different populations and conditions.

## Toxicity and tolerance

Despite the numerous physiological effects of dietary flavonoids in the body, excessive consumption can potentially lead to liver damage. Consuming high-dose green tea extracts (843  mg/day EGCG) or placebo capsules for 3, 6, 9, 12  months in postmenopausal women participants, ALT is always below 30, reflecting “healthy” ALT concentrations. However, compared from baseline to months 6 and 9, ALT significantly elevated by 26.4 %-46 % [285]. The influence of uridine 5'-diphospho-glucuronosyltransferase 1A4 (UGT1A4) genotypes on changes in liver injury biomarkers was also investigated. The UGT1A4 (rs6755571) A/C genotype showed a mean ALT change of + 78.1 % and + 82.1 % from baseline to months 6 and 9, respectively, while the C/C genotype showed a change of + 28.0 % and + 30.1 %. The A/C genotype of UGT1A4 (rs6755571) could be a significant risk factor for elevated blood transaminase levels after 6–9 months intake of 843  mg/day EGCG [285]. A daily dose of 714 mg of green tea flavonoids for 3 weeks is considered safe for healthy men [286]. Repeated administration of 800 mg EGCG for 4 weeks did not result in any discernible alterations in blood counts and blood chemical profiles [284]. Consumption of 1.3 g of tea flavonoids (equivalent to 800 mg of EGCG) over 3 weeks did not result in any increase in liver enzymes in prostate cancer patients [287]. Administration of 50 mg of ellagitannin and 10 mg of ellagic acid for 4 weeks did not result in any notable toxicity [288]. While EGCG is generally well tolerated, doses exceeding 1200 mg have been linked to hepatotoxic consequences in certain patients. In a clinical trial involving 47 individuals with multiple system atrophy, with an average age of 60 years, two patients in the EGCG group had to discontinue treatment due to hepatotoxicity after receiving 1200 mg/day for 48 weeks [282]. A group of 15 patients, all over 40 years old, with rectal abnormal crypt foci were randomly assigned to receive oral Poly E (780 mg EGCG) daily for 6 months. Although Poly E was generally well-tolerated and did not cause severe toxicity, one participant in the Poly E group experienced adverse effects including increased levels of hepatic transaminase [274]. A separate study found that the dose-limiting toxicity rate at 600 mg of Poly E for 6 months in women was 27 % [289]. Administration regimen was composed of 400 mg EGCG for 4 weeks, followed by 800 mg of EGCG for another 4 weeks, and finally 1200 mg of EGCG for another 4 weeks. Compared to the placebo group, the epigallocatechin gallate group had a greater incidence of hepatotoxic effects, highlighting the significance of closely monitoring liver enzymes and avoiding doses of EGCG above 1200 mg [282]. Elevated levels of bilirubin were seen in the patient who consumed 3.9 g of silibinin. An 8-week administration of 3.9 g silibinin resulted in increased levels of ALT and aspartate transferase (AST) in a prostate cancer patient [290]. Overall, dietary flavonoids do not generally induce liver damage, but intake exceeding 1000 mg per day warrants careful consideration.

## Future perspective and challenges for the use of flavonoids

Several clinical investigations have shown that the lack of efficacy of flavonoids contrasts with compelling evidence derived from rodent models, thereby casting question on the rationale behind using flavonoids as a human nutritional supplement for metabolic disorders in human. Current review systematically summarizes the potential targets through which flavonoids may influence general metabolism, nutrients digestion and absorption, intestinal homeostasis, gut microbiota, endothelial cells, the liver, and other organs. The presence of interindividual diversity in the absorption and metabolism of flavonoids can lead to diverse responses to these compounds. Population characteristics such as age, gender, sex, BMI, health status, gut microbiota composition, and genetic polymorphisms may account for these phenomena [291]. The intestinal microbiota significantly influences the variations across individuals in the metabolism of the majority of flavonoids. The swift changes in microbiome composition and function due to dietary modifications occurring within 1 to 4 days complicate the study of the benefits of flavonoids in human [292]. Some phenolic sub-classes may also be influenced by genetic variations in enzymes involved in polyphenol metabolism [293]. Furthermore, many studies we referenced have a limited number of human participants (Table 2, Table 3), highlighting the need for larger-scale research. Indeed, the intricate nature of flavonoid species might result in varied impacts on the body, leading to diverse findings. This article endeavors to provide a comprehensive overview of the essential roles of flavonoids in the population and briefly summarizes the underlying mechanisms based on murine and *in vitro* studies; however, it exhibits certain deficiencies. Firstly, there may be omissions in the literature collection. Secondly, some mechanisms related to metabolic abnormalities, such as glucose metabolism in organs like the pancreatic islets, have not been addressed.

**Fig. 6:**
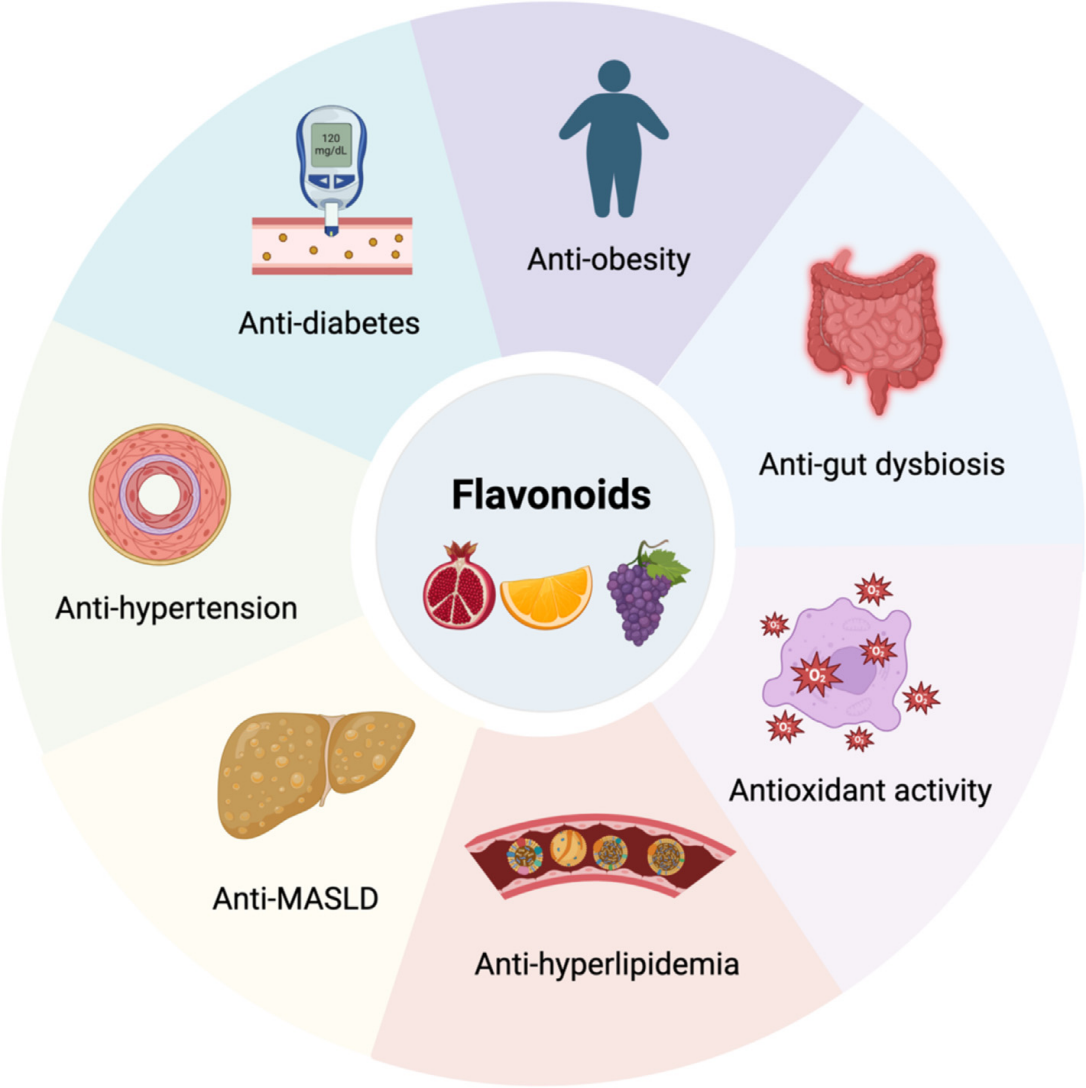
Disease prevention of flavonoids in humans.

Epidemiological and clinical data provide valuable insights into the health benefits of dietary flavonoids, facilitating the development of evidence-based dietary guidelines for their application in disease prevention and health promotion (Fig. 6). The following are our insights:

(1) Regarding the health mechanisms of flavonoids in the human body, there are still a lot of unanswered questions. For example, which is more crucial in gastrointestinal tract: the reconfiguration of the gut microbiota, the newly produced metabolites, or both? Flavonoids influence the homeostasis of peripheral organs, including the liver, muscle, and adipose tissue, through which specific pathways, and whether potentially mediated by the flavonoids itself, its metabolites, or other unknown signaling messengers?

(2) While higher intake of flavonoids is generally beneficial for health, it is crucial to consider specific populations. Individuals with iron deficiency or those at a heightened risk of iron insufficiency, such as young children and pregnant women, should be mindful of the flavonoids they intake. Simultaneously, it is also important to consider the dosage, as an excessive consumption of flavonoids can lead to liver injury.

(3) Flavonoids can complement medications for hypertension and hyperlipidemia by enhancing endothelial function, lowering blood pressure, and reducing LDL cholesterol levels, thereby promoting cardiovascular health. Nevertheless, they should not be regarded as substitutes for traditional medical therapies.

(4) The wide range of varieties and complexity of a diet rich in flavonoids enhance the associated health benefits. However, one research recently found antagonistic effect of kale soluble dietary fiber and kale flavonoids fails to alleviate colitis [294], suggesting the complexity of nutrient interactions. Generally, adopting a diverse diet guarantees a comprehensive intake of various flavonoids, optimizing their combined health benefits. Consequently, the consumption of colored fruits and vegetables is recommended in everyday routines.

## Compliance with Ethics Requirements

*Neither human nor animal experiments were conducted*.

## CRediT authorship contribution statement

**Xiaopeng Li:** Conceptualization, Data curation, Writing – original draft, Supervision, Project administration, Funding acquisition. **Enjun Xie:** Writing – review & editing, Visualization. **Shumin Sun:** Writing – review & editing, Visualization. **Jie Shen:** Writing – review & editing. **Yujin Ding:** Visualization. **Jiaqi Wang:** Writing – review & editing, Funding acquisition. **Xiaoyu Peng:** Writing – review & editing. **Ruting Zheng:** Writing – review & editing. **Mohamed A. Farag:** Writing – review & editing. **Jianbo Xiao:** Conceptualization, Writing – review & editing, Resources, Project administration.

## Funding

This work was supported by grants from the National Key R&D Program of China (2023YFD2100302), the National Natural Science Foundation of China (32200961), The Science and Technology Innovation Program of Hunan Province (2024RC3239), the Natural Science Foundation of Hunan Province (2023JJ40365), the Hunan Provincial Agricultural Science and Technology Innovation Fund Project (2023CX111), and Hunan Engineering and Technology Research Center for Nutrition and Health Products Innovation Platform and Talent Plan (2019TP2066).

## Declaration of competing interest

*The authors declare that they have no known competing financial interests or personal relationships that could have appeared to influence the work reported in this paper*.
